# CAD-CAM vs. conventional denture bases: a systematic review with network meta-analysis of *in vitro* studies comparing strength, hardness, toughness, and elastic properties

**DOI:** 10.3389/fdmed.2025.1638794

**Published:** 2025-08-11

**Authors:** Rohit Kunnath Menon, Hui Xin Yew, Benjamin Chen Tze Wei, Farah Mohammed Ramadan, Afraa Ibrahim Soliman, Sajesh Veettil

**Affiliations:** ^1^ College of Dentistry, Ajman University, Ajman, United Arab Emirates; ^2^ School of Dentistry, IMU University, Kuala Lumpur, Malaysia; ^3^ Department of Pharmacy Practice, School of Pharmacy, IMU University, Kuala Lumpur, Malaysia

**Keywords:** CAD-CAM, digital dentures, milled, 3D-printed, denture, *in vitro*

## Abstract

**Background:**

Scientific evidence from *in vitro* studies comparing the mechanical properties of dentures fabricated with computer-aided design-computer-aided manufacturing (CAD-CAM) and conventional techniques is inconclusive. This systematic review with meta-analysis was conducted to analyze the current evidence comparing the mechanical properties of conventional and digitally fabricated denture bases from *in vitro* studies.

**Materials and methods:**

A systematic search was conducted in PubMed, Scopus, and Medline for *in vitro* studies from inception until 16 January 2025. The review had been registered with the International Prospective Register of Systematic Reviews PROSPERO: CRD42024531425). A network meta-analysis compared conventional and digitally fabricated denture bases’ flexural strength, hardness, flexural modulus, elastic modulus, impact strength, fracture toughness, yield point, and toughness. Risk of bias was assessed by using RoBDEMAT (RoB 2.0).

**Results:**

4,994 articles were identified, 966 duplicates were removed, 3,971 were excluded by title and abstract screening, 57 were assessed by full-text reading, and 42 were included in the quantitative synthesis. As per the sensitivity analysis performed after excluding low-quality studies, the network meta-analysis results indicate that milled digital denture bases exhibit higher flexural strength [SMD = 2.13 (95% CI: 0.21, 4.05)] compared to 3D-printed digitally fabricated denture bases. Bias incorporated from higher values from one study diminishes the quality of evidence for impact strength and flexural modulus.

**Conclusion:**

Milled digital denture bases exhibit superior flexural strength to 3D-printed and conventionally fabricated denture bases under laboratory conditions. High-quality *in vitro* studies are recommended to provide conclusive evidence for other mechanical properties.

**Systematic Review Registration:**

PROSPERO CRD42024531425.

## 1 Introduction

Complete removable dentures are used to rehabilitate edentulous patients. In contrast to chrome-cobalt removable partial dentures used to rehabilitate partial edentulism, complete dentures lack retentive and stabilizing components that may be used to gain retention and support from remaining abutments (1). Consequently, the material and technique employed to fabricate complete dentures are critical and influence the mechanical properties and clinical performance. Complete dentures may be fabricated using conventional or digital processing techniques.

The conventional technique utilizes polymethylmethacrylate (PMMA) resin. PMMA entails low cost, acceptable aesthetics, ease of repair, and handling characteristics (2). However, the manufacturing process introduces internal stresses, polymerization shrinkage, and dimensional variations, affecting accuracy and retention (3). The CAD-CAM dentures manufacturing technique simplifies the clinical and laboratory protocols with reduced appointments and minimal dimensional variations (4).

The subtractive technique involves designing the prosthesis in virtual CAD software. The chosen geometry is achieved by machining following the digital model. A pre-polymerized resin block is milled, followed by prefabricated or milled denture teeth bonding (5). Additive manufacturing involves the layer-by-layer build-up of the prosthesis, circumventing any limitations in the geometrical design of the envisioned prosthesis. This method reduces excess material consumption but is restrained by technical limitations (6).

Manufacturing methods such as milling (Subtractive) and 3D printing (Additive) influence the mechanical properties. The milled (subtractive) method is fabricated from pre-polymerized polymethyl methacrylate (PMMA) at high temperature and pressure, promising minimal residual monomer, adequate hygienic outcomes, and enhanced mechanical properties (7). In contrast, the additive method utilizes photopolymerized resin, heavily relying on the parameters used during printing and subsequent curing procedures (8). Digital denture fabrication has been associated with achieving fewer visits, appointments, and manufacturing time, in addition to better mechanical properties (9, 10). Denture bases are subjected to shear, compressive, and tensile stresses during clinical function. Damage and dimensional variations may be minimized by ensuring adequate hardness (11). Plastic deformation and satisfactory functional performance may be ensured by providing adequate flexural strength, impact strength, and yield point (12, 13). Impact strength is influenced by manufacturing method, stress concentration, material used, thermal factors, specimen geometry, and position (14, 15). A recent meta-analysis has compared the mechanical properties of denture bases fabricated with digital and conventional techniques (16). However, only pairwise comparisons were included. Network meta-analysis (NMA) augments conventional pairwise meta-analysis, where only two interventions are compared by combining manifold evidence sources derived from a network of studies comparing multiple interventions. NMA enables investigators to combine direct and indirect evidence to establish comparative efficacy and acceptability across studies of all denture base types. The purpose of this NMA was to compare the flexural strength, hardness, flexural modulus, elastic modulus, impact strength, fracture toughness, yield point, and toughness between denture bases fabricated by conventional and digital techniques. The null hypothesis was that no difference would be found in flexural strength, hardness, flexural modulus, elastic modulus, impact strength, fracture toughness, yield point, and toughness between conventional and digital denture bases as evaluated in *in vitro* studies.

## 2 Material and methods

### 2.1 Search strategy and inclusion criteria

A systematic review of *in vitro* studies compared the mechanical properties of digitally and conventionally fabricated denture bases. The protocol for the systematic review was registered with the International Prospective Register of Systematic Reviews [PROSPERO: (CRD42024531425)] and reported according to the Preferred Reporting Items for Systematic Reviews and Meta-Analysis (PRISMA) extension statement of NMA (17) (Supplementary Tables 1, 2, available online). Relevant studies were identified from 3 databases, PubMed, Scopus, and Medline, from inception to 16 January 2025. In addition to three databases, references from previous systematic reviews and grey literature were thoroughly searched (Supplementary Table 3). *In vitro* studies were included, and clinical comparisons, editorials, consensus or clinical conferences, and case reports were excluded. The criteria were based on the population, intervention, comparison, and outcome (PICO) strategy.

#### 2.1.1 Population

Complete denture bases were fabricated in the laboratory using polymethylmethacrylate.

#### 2.1.2 Intervention

Digital denture bases are fabricated either by milling or 3D printing.

#### 2.1.3 Comparator

Complete denture bases fabricated by a conventional technique, including compression molding, injection molding, or autopolymerization.

#### 2.1.4 Outcomes

Flexural strength, hardness, flexural modulus, elastic modulus, impact strength, fracture toughness, yield point, and toughness.

### 2.2 Data extraction and quality assessment

Titles and abstracts were independently screened by two reviewers (A.I.S., F.M.R.) for eligible studies, followed by full-text reading. Ineligible studies were excluded, and the reasons for exclusion were documented. The two reviewers extracted data independently and in duplicate into a data extraction form. Disagreements and discrepancies were resolved by discussion with a third reviewer (R.K.M.). The risk of bias within each study was independently assessed by two reviewers (YHX, BCTW) using RoBDEMAT (RoB 2.0) (18). Disagreements and discrepancies were resolved by 2.1.2 discussion with a third reviewer (R.K.M).

### 2.3 Data synthesis and statistical analysis

Standardized mean differences (SMD) and 95% confidence intervals were used as summary statistics for continuous outcomes. A standard pairwise meta-analysis was performed using a random-effects (DerSimonian and Laird) model for direct comparisons (19). If a direct comparison was based on two or more studies, heterogeneity among trials was assessed by considering the *I*^2^ statistics (20). To synthesize the available evidence by combining direct and indirect evidence from different studies, a random-effects NMA was applied (21–23). The probability of each denture type being the best was estimated by constructing rankograms and their surface area under the cumulative ranking (SUCRA). (24, 25). A comparison-adjusted funnel plot was used to examine the publication bias. Local inconsistency in the network was assessed using node-splitting models, which compare direct and indirect evidence for specific treatment comparisons (26). Sensitivity analysis by excluding low-quality studies was performed. Studies that reported 'Sufficiently Reported' or “Adequate” for more than 80% of applicable criteria in the RoBDEMAT (RoB 2.0) framework were classified as “low risk of bias”, and the remaining studies were classified as “high risk of bias”. A statistical software program (Stata version 15.0; StataCorp) was used for statistical analysis and graph generation (24).

## 3 Results

**Figure 1:**
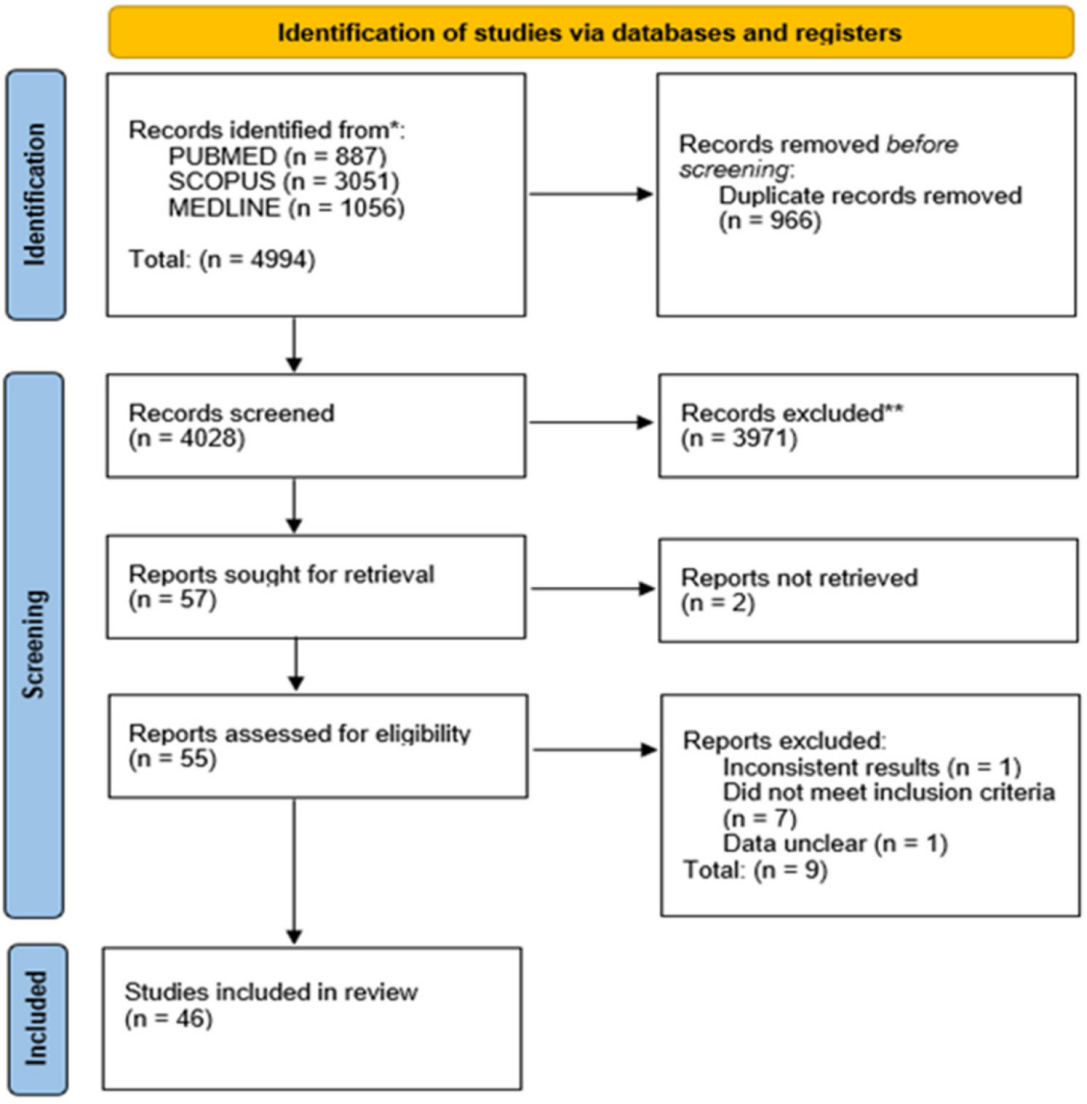
PRISMA flow diagram summarising the study selection process for eligible trials.

A total of 4,994 articles were identified, of which 966 duplicates were removed, and 3,971 were excluded by screening the titles and abstracts. A total of 57 articles were assessed by full-text reading; 46 articles (27–71 were selected in the qualitative synthesis, and 4 articles 27, 33, 44, 69 were excluded as quantitative data were not obtained for analysis on the relevant outcomes. 42 articles (7, 27–67) were included in the quantitative synthesis. The preferred reporting items for systematic reviews and meta-analyses (PRISMA) flow diagram is depicted in Figure 1; Supplementary Table 4 shows the characteristics of the studies included. The quality assessment of each study using the RoBDEMAT assessment tool is provided in Supplementary Table 5. Milled digital denture bases (MIL), 3D-printed digital denture bases (TDP), conventional compression molded denture bases (CCM), conventional injection molded denture bases (CCI), and traditional auto-polymerized denture bases (CCA) were compared.

**Figure 2:**
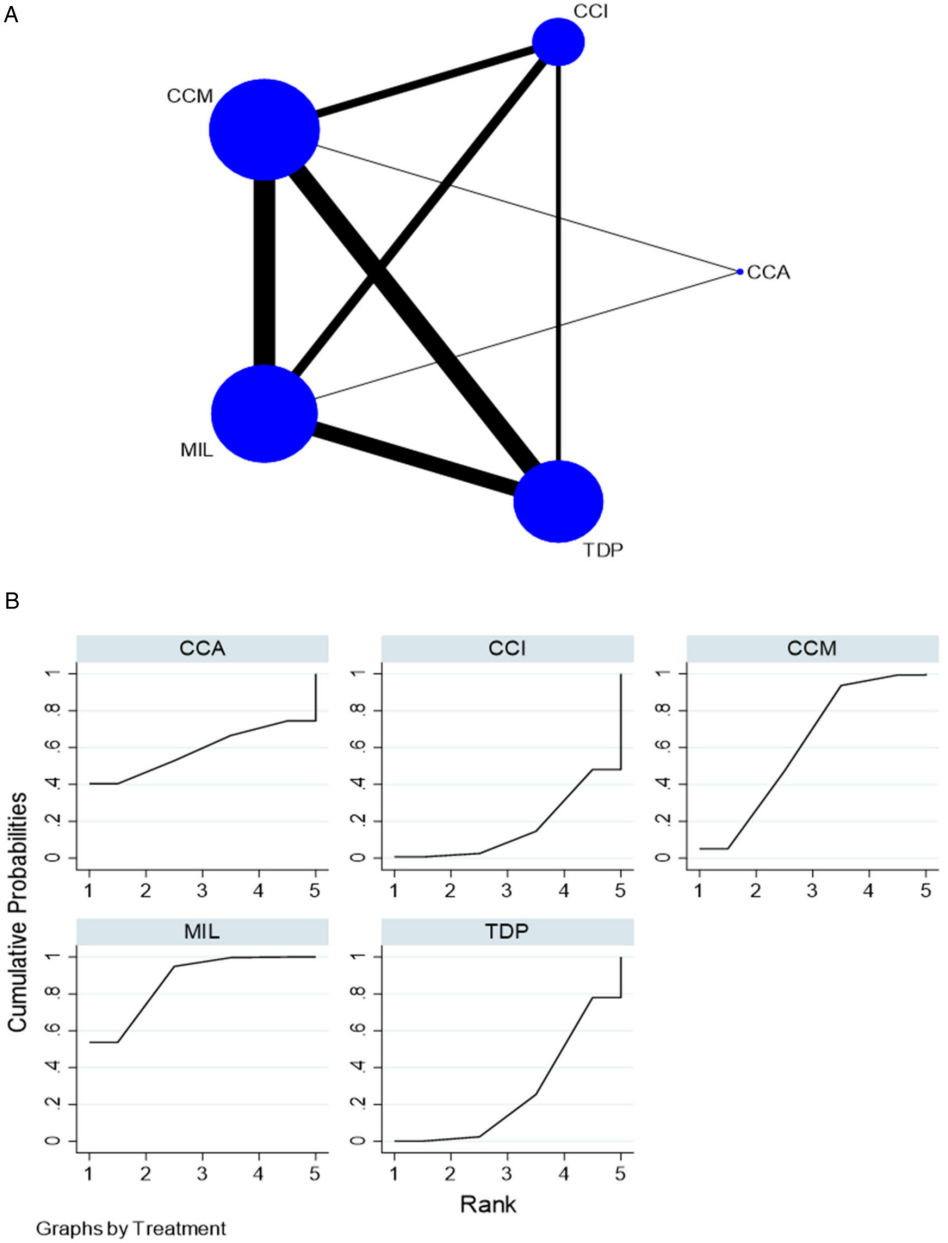
**(A)** Network plot for flexural strength. CCM, conventional compression moulding; CCI, conventional injection moulding; CCA, conventional autopolymerisation; MIL, CAD-CAM milled; TDP, three-dimensional printed. **(B)** SUCRA ranking curve for flexural strength.

For flexural strength, 35 *in vitro* studies comparing five interventions were included in the NMA as seen in Figure 2A. MIL demonstrated higher flexural strength than TDP [SMD = 1.62 (95% CI: 0.48, 2.76)] *P* < 0.05 and CCI [SMD = 2.03 (95% CI: 0.50, 3.56)] *P* < 0.05. Supplementary Table 6 summarizes the SMD and the ranking of the interventions, while Figure 2B shows the SUCRA ranking curves for each intervention in the network. MIL ranked the highest, followed by CCM, CCA, TDP, and CCI. The results of the pairwise meta-analysis are depicted in the forest plot as seen in Supplementary Figure 1. The network and pairwise estimates for all the interventions are summarized in Table 1. Based on the comparison-adjusted forest plots, publication bias could be detected as seen in Supplementary Figure 2. Based on the global inconsistency test (*p* = 0.12), no significant inconsistency was detected. Sensitivity analysis, excluding low-quality studies, was performed. MIL demonstrated significantly higher flexural strength than TDP (SMD = 2.13 [95% CI: 0.21, 4.05). The results of the sensitivity analysis are available in Supplementary Table 7.

**Table 1:** League table showing the network and pairwise results for flexural strength.

**CCA**	NA	−2.93 (−4.23, −1.63)*	NA	1.54 (0.53, 2.56)*
*1.35 (−3.41, 6.11)*	**CCI**	−1.94 (−1.05, −2.83)*	−0.82 (−2.08, 0.44)	−1.67 (−3.10, −0.24)*
*−0.68 (−5.25, 3.89)*	*−2.03 (−3.56, −0.50)* *	**MIL**	0.96 (1.59, 0.32)*	1.24 (0.42, 2.05)*
*0.94 (−3.70, 5.59)*	*−0.41 (−2.03, 1.22)*	*1.62 (0.48, 2.76)* *	**TDP**	−1.19 (−1.92, −0.45)*
*0.02 (−4.55, 4.59)*	*−1.33 (−2.86, 0.21)*	*0.70 (−0.37, 1.77)*	*−0.92 (−2.02, 0.18)*	**CCM**

CCM, conventional compression moulding; CCI, conventional injection moulding; CCA, conventional autopolymerisation; MIL, CAD-CAM milled; TDP, three-dimensional printed.

The bold fonts depict different groups.

* Indicates *P* < 0.05, Network results are denoted in italics.

**Figure 3:**
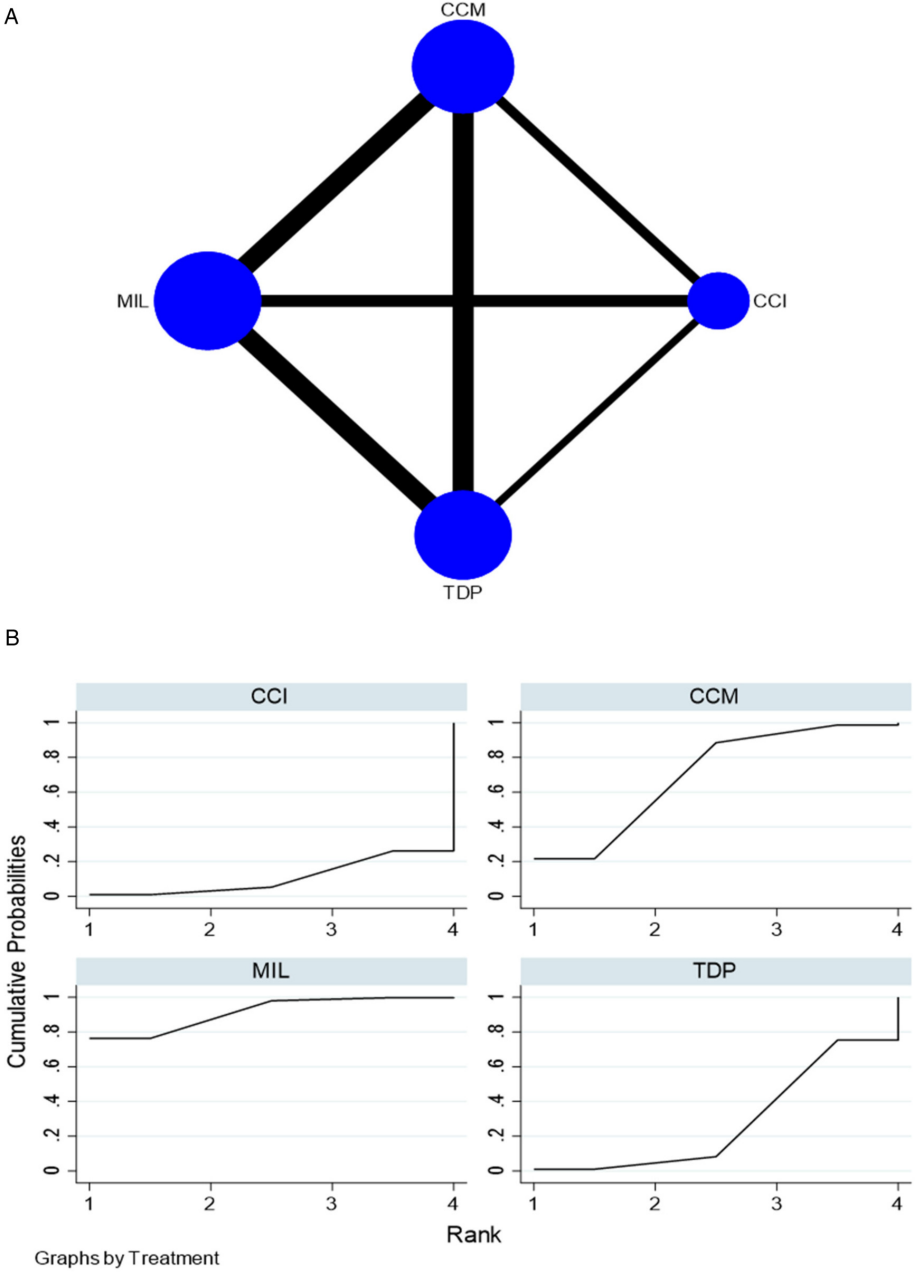
**(A)** Network plot for hardness. **(B)** SUCRA ranking curve for hardness. CCM, conventional compression moulding; CCI, conventional injection moulding; MIL, CAD-CAM milled; TDP, three-dimensional printed.

For hardness, 16 *in vitro* studies comparing four interventions were included in the NMA, as seen in Figure 3A. MIL demonstrated higher hardness than CCI [SMD = 4.06 (95% CI: 0.51, 7.62)] *P* < 0.05 and TDP [SMD = 2.87 (95% CI: 0.14, 5.60)] *P* < 0.05. Supplementary Table 8 summarizes the SMD and the ranking of the interventions, while Figure 3B shows the SUCRA ranking curves for each intervention in the network. MIL ranked the highest, followed by CCM, TDP, and CCI. The results of the pairwise meta-analysis are depicted in the forest plot as seen in Supplementary Figure 3. The network and pairwise estimates for all the interventions are summarized in Table 2. Based on the comparison-adjusted forest plots, publication bias could be detected as seen in Supplementary Figure 4. Based on the global inconsistency test (*p* = 0.052), no significant inconsistency was detected. Sensitivity analysis, excluding low-quality studies, revealed no significant differences. The results of the sensitivity analysis are available in Supplementary Table 9.

**Table 2:** League table showing the network and pairwise results for hardness.

**CCI**	−2.74 (−6.31, 0.82)	−2.53 (−7.43, 2.37)	−5.99 (−10.28, −1.70)*
*−4.06 (−7.62, −0.51)* *	**MIL**	2.91 (1.35, 4.47)*	0.42 (−1.18, 2.01)
*−1.19 (−4.92, 2.54)*	*2.87 (0.14, 5.60)* *	**TDP**	−2.26 (−3.60, −0.92)*
*−3.07 (−6.71, 0.57)*	*0.99 (−1.73, 3.72)*	*−1.88 (−4.58, 0.83)*	**CCM**

CCM, conventional compression moulding; CCI, conventional injection moulding; MIL, CAD-CAM milled; TDP, three-dimensional printed.

The bold fonts depict different groups.

* Indicates *P* < 0.05, network results are denoted in italics.

**Figure 4:**
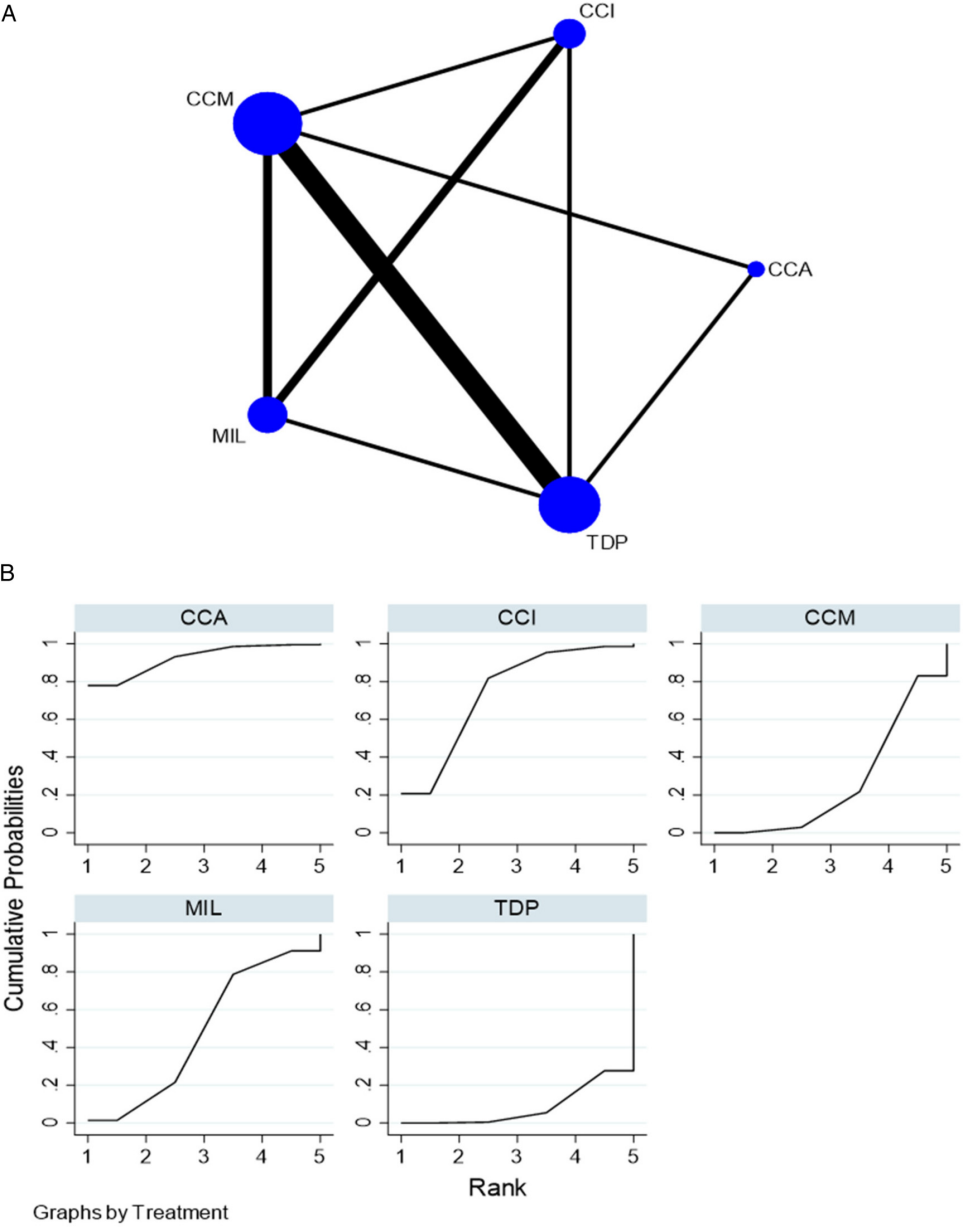
**(A)** Network plot for impact strength. CCM, conventional compression moulding; CCI, conventional injection moulding; CCA, conventional autopolymerisation; MIL, CAD-CAM milled; TDP, three-dimensional printed. **(B)** SUCRA ranking curve for impact strength.

For impact strength, 8 *in vitro* studies comparing five interventions were included in the NMA, as seen in Figure 4A. CCA demonstrated higher impact strength when compared with CCM [SMD = 8.88 (95% CI: 1.17, 16.59)] *P* = 0.024 and TDP [SMD = 10.25 (95% CI: 2.56, 17.93)] *P* < 0.05. CCI demonstrated higher impact strength when compared with TDP [SMD = 6.25 (95% CI: 0.11, 12.38)], *P* < 0.05. Supplementary Table 10 summarizes the SMD and the ranking of the interventions, while Figure 4B shows the SUCRA ranking curves for each intervention in the network. CCA ranked the highest, followed by CCI, MIL, CCM, and TDP. The results of the pairwise meta-analysis are depicted in the forest plot as seen in Supplementary Figure 5. The network and pairwise estimates for all the interventions are summarized in Table 3. Based on the comparison-adjusted forest plots, publication bias could be detected in Supplementary Figure 6. Based on the global inconsistency test (*p* = 0.42), no significant inconsistency was detected. Sensitivity analysis, excluding low-quality studies, was performed. CCA demonstrated significantly higher impact strength than TDP (SMD = 10.29 [95% CI: 1.15, 19.44). The results of the sensitivity analysis are available in Supplementary Table 1^1^.

**Table 3:** League table showing the network and pairwise results for impact strength.

**CCA**	NA	NA	6.49 (5.08, 7.90)*	16.97 (13.53, 20.41)*
*4.00 (−5.46, 13.46)*	**CCI**	2.60 (−4.64, 9.84)	9.22 (7.47, 10.97)*	1.95 (1.07, 2.83)*
*6.75 (−2.22, 15.72)*	*2.75 (−3.00, 8.51)*	**MIL**	4.28 (3.35, 5.21)*	2.31 (−0.47, 5.09)
*10.25 (2.56, 17.93)* *	*6.25 (0.11, 12.38)* *	*3.49 (−1.94, 8.93)*	**TDP**	−0.81 (−3.55, 1.93)
*8.88 (1.17, 16.59)* *	*4.88 (−1.07, 10.83)*	*2.13 (−2.90, 7.16)*	*−1.36 (−4.91, 2.19)*	**CCM**

CCM, conventional compression moulding; CCI, conventional injection moulding; CCA, conventional autopolymerisation; MIL, CAD-CAM milled; TDP, three-dimensional printed.

The bold fonts depict different groups.

* Indicates *P* < 0.05, network results are denoted in italics.

**Figure 5:**
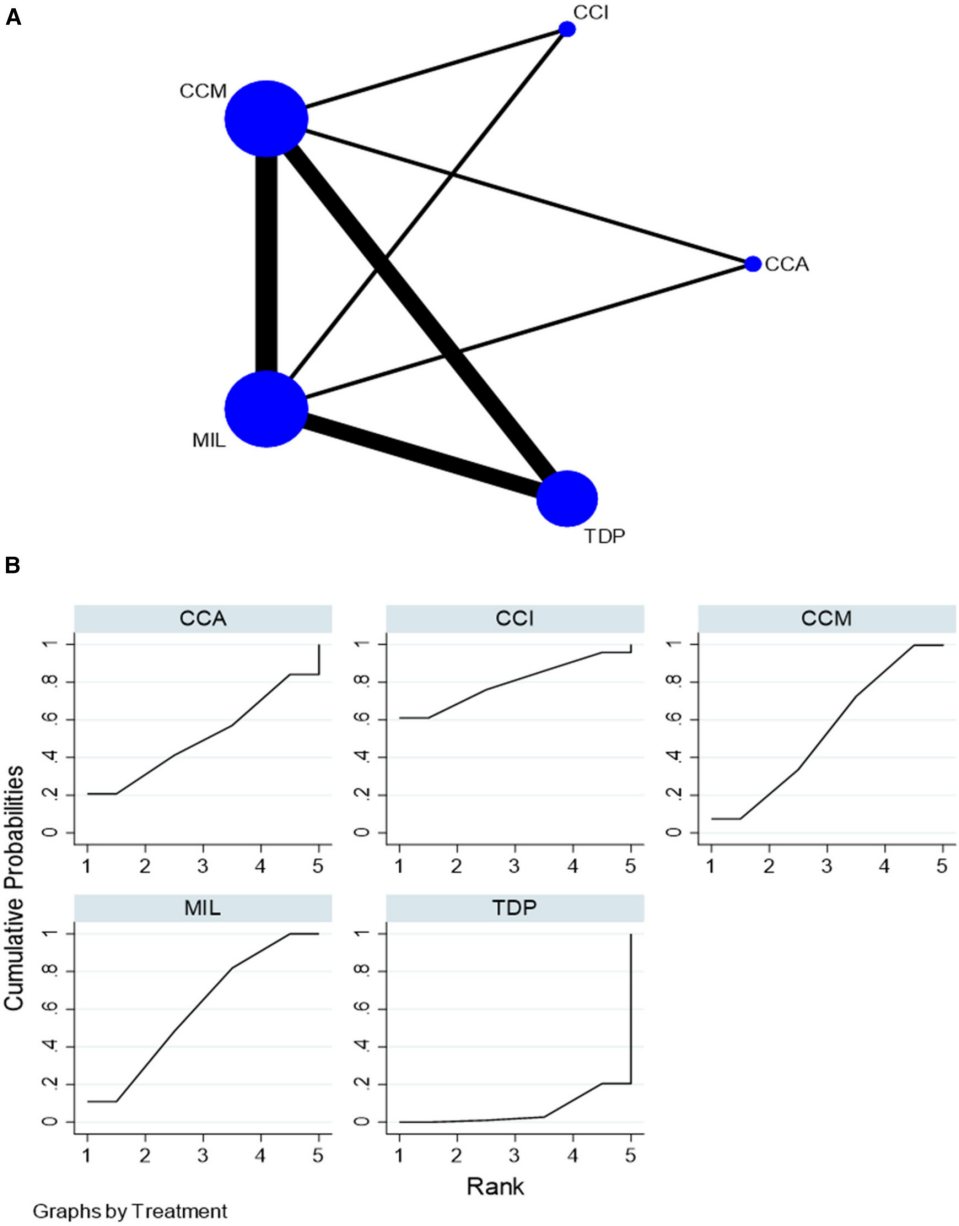
**(A)** Network plot for elastic modulus. CCM, conventional compression moulding; CCI, conventional injection moulding; CCA, conventional autopolymerisation; MIL, CAD-CAM milled; TDP, three-dimensional printed. **(B)** SUCRA ranking curve for elastic modulus.

For elastic modulus, seven *in vitro* studies comparing five interventions were included in the NMA as seen in Figure 5A. MIL demonstrated higher elastic modulus than TDP [SMD = 5.54 (95% CI: 0.75, 10.)] *P* < 0.05, and CCM demonstrated higher elastic modulus than TDP [SMD = 5.16 (95% CI: 0.39, 9.92)] *P* = 0.034. Supplementary Table 1^2^ summarizes the SMD and the ranking of the interventions, while Figure 5B shows the SUCRA ranking curves for each intervention in the network. CCI ranked the highest, followed by MIL, CCM, CCA, and TDP. The results of the pairwise meta-analysis are depicted in the forest plot as seen in Supplementary Figure 7. The network and pairwise estimates for all the interventions are summarized in Table 4. Based on the comparison-adjusted forest plots, publication bias could be detected in Supplementary Figure 8. Based on the global inconsistency test (*p* = 0.93), no significant inconsistency was detected. Sensitivity analysis, excluding low-quality studies, revealed no significant differences. The results of the sensitivity analysis are available in Supplementary Table 1^3^.

**Table 4:** League table showing the network and pairwise results for elastic modulus.

**CCA**	NA	−1.10 (−1.80, −0.41)*	NA	−0.48 (−1.37, 0.41)
*−3.80 (−16.03, 8.43)*	**CCI**	3.99 (3.11, 4.88)*	NA	1.77 (1.17, 2.37)*
*−0.85 (−9.75, 8.06)*	*2.95 (−5.95, 11.86)*	**MIL**	3.57 (0.99, 6.15)*	0.65 (−1.40, 2.69)
*4.69 (−4.96, 14.34)*	*8.49 (−1.15, 18.14)*	*5.54 (0.75, 10.33)* *	**TDP**	−4.66 (−7.72, −1.61)*
*−0.46 (−9.37, 8.44)*	*3.34 (−5.56, 12.24)*	*0.38 (−3.88, 4.64)*	−5.16 (−9.92, −0.39)*	**CCM**

CCM, conventional compression moulding; CCI, conventional injection moulding; CCA, conventional autopolymerisation; MIL, CAD-CAM milled; TDP, three-dimensional printed.

The bold fonts depict different groups.

* Indicates *P* < 0.05, network results are denoted in italics.

**Figure 6:**
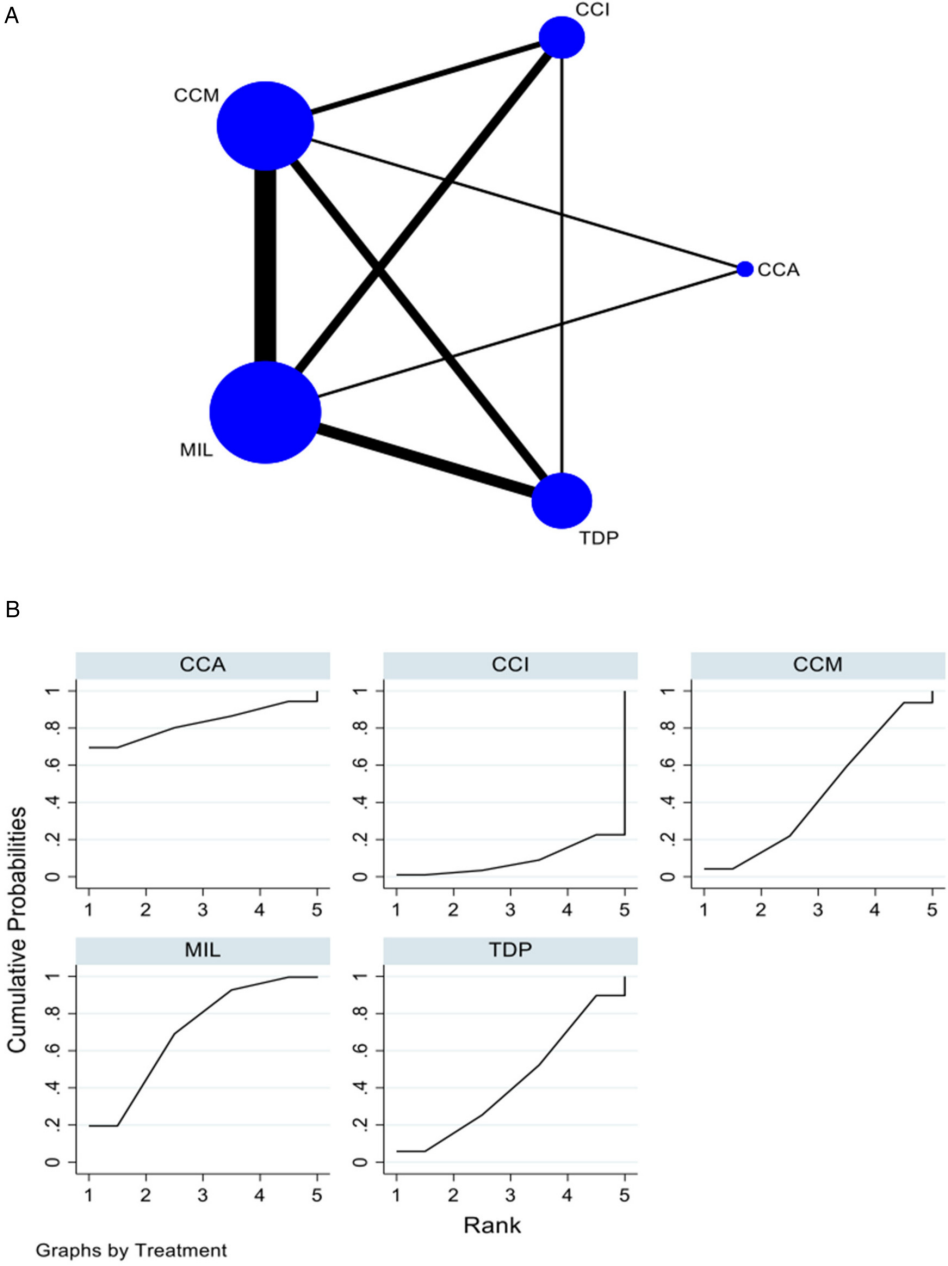
**(A)** Network plot for flexural modulus. CCM, conventional compression moulding; CCI, conventional injection moulding; CCA, conventional autopolymerisation; MIL, CAD-CAM milled; TDP, three-dimensional printed. **(B)** SUCRA ranking curve for flexural modulus.

For flexural modulus, 12 *in vitro* studies comparing five interventions were included in the NMA as seen in Figure 6A. In the primary analysis, none of the interventions demonstrated significant results. Supplementary Table 14 summarizes the SMD and the ranking of the interventions, while Figure 6B shows the SUCRA ranking curves for each intervention in the network. CCA ranked the highest, followed by MIL, CCM, TDP, and CCI. The results of the pairwise meta-analysis are depicted in the forest plot as seen in Supplementary Figure 9. The network and pairwise estimates for all the interventions are summarized in Supplementary Table 15. Based on the comparison-adjusted forest plots, publication bias could be detected in Supplementary Figure 10. Based on the global inconsistency test (*p* = 0.95), no significant inconsistency was detected. Sensitivity analysis, excluding low-quality studies, was performed. CCI demonstrated significantly higher flexural modulus when compared with MIL [SMD = 3.54 (95% CI: 0.97, 6.12)] *P* < 0.05, TDP [SMD = 4.38 (95% CI: 1.47, 7.28)] and CCM [SMD = 2.69 (95% CI: 0.14, 5.24)] *P* < 0.05. The results of the sensitivity analysis are available in Supplementary Table 16.

None of the interventions demonstrated significant results for fracture toughness or yield point. Only two articles contributed to the included data for strain at the yield point and toughness. The network plots, SUCRA ranking curves, forest plots, and funnel plots are provided in Supplementary Figures 11–22. SMD, ranking of interventions, network, and pairwise estimates are provided in Supplementary Tables 17–24. Sensitivity analysis was not performed for fracture toughness, yield point, strain at yield point, and toughness because there was only one trial after excluding all low-quality studies.

Node-splitting analysis was performed to assess local inconsistency in the network as depicted in Supplementary Tables 25–29. Significant local inconsistency was not identified for any of the important findings in the primary and sensitivity analyses, indicating no significant inconsistency between direct and indirect evidence across the network. The PRISMA checklist for this review is provided as Supplementary Table 30.

## 4 Discussion

The null hypothesis that no differences would be found in the mechanical properties of conventional and digitally fabricated denture bases was rejected. The highest bending stress expressed in a material at the moment of fracture is termed flexural strength (68). Denture bases may fail due to flexure exhibited during mastication. High flexural strength prevents catastrophic fatigue failure (69–71). Flexural strength is conventionally measured by a 3-point test evaluating denture bases' resistance and stiffness (72–74). As per the NMA results, MIL demonstrated significantly higher flexural strength than TDP and CCI. No significant differences were observed between other denture base types. Ranking of the denture bases per the SUCRA ranking curve corroborates this result, with MIL being ranked as the highest. Pairwise results confirm this finding. Individual pairwise comparisons have indicated more significant differences between denture base types. However, a combination of direct and indirect evidence with the NMA corroborates the evidence that MIL demonstrates a higher flexural strength than TDP. This finding aligns with previous research where 3D-printed materials have demonstrated lower flexural strength (45, 75). Reinforcement of 3D-printed filler resins has been suggested to improve strength (76–78). However, this effort may prove counterintuitive as fillers may detrimentally affect the flexural strength if proper concentrations are not maintained (44). The printing orientation influences the flexural strength, with a horizontal printing orientation at 300 demonstrating the highest flexural strength (79, 80). A Lesser thickness of layers has been associated with higher flexural strength (81). The platform's printing position also impacts the flexural strength (82). The type of photoinitiator used for curing, post-curing time, temperature, post-curing rinse, and curing method influence the flexural strength (83–85). Consequently, controlling the composition and printing parameters is critical to circumvent the negative consequences of reduced flexural strength in 3-D printed dentures.

Hardness is measured by the resistance to localized plastic deformation induced by mechanical indentation (75). Higher hardness values reduce microbial adhesion and provide color stability to the denture base (86). As per the NMA results, MIL demonstrated significantly higher values for hardness than TDP and CCI. Ranking of the denture bases per the SUCRA ranking curve corroborates this result, with MIL being ranked the highest. Pairwise meta-analysis results are concordant. Previous meta-analysis, which summarized only the direct evidence, did not establish the superior hardness of milled dentures, finding no significant differences among all denture types (16). The higher hardness of MIL may be attributed to higher processing temperature and pressure, which diminishes the residual monomer and plasticity (87). Hence, MIL denture bases will exhibit reduced susceptibility to localized plastic deformation by abrasion or indentation. Further, milled dentures have been shown to be more stable to changes in color and hardness after prolonged exposure to denture cleansers (88). The longevity of 3-D printed and conventional dentures may also be diminished due to mechanical insults leading to plaque retention and subsequent pigmentations.

Elastic modulus is defined as the stiffness of a material and can be calculated as the ratio of elastic stress to elastic strain (12). As per the NMA results, both MIL and CCM demonstrated significantly higher values for elastic modulus than TDP. Ranking of the denture bases per the SUCRA ranking curve ranks CCI as the highest, followed by MIL. This is corroborated by the pairwise results where only CCI demonstrated significantly higher elastic modulus than MIL. MIL demonstrated significantly higher elastic modulus than CCA and TDP. A previous meta-analysis, which summarized only the direct evidence, did not establish the superior elastic modulus of milled dentures compared to conventional dentures (16). Considering that there was only one study directly comparing CCI vs. MIL, the results comparing these entities may be affected by significant bias. A combination of direct and indirect evidence with the NMA supports the evidence that MIL has significantly higher elastic modulus when compared with TDP. Consequently, MIL denture bases may be more resistant to permanent deformation and wear when exposed to masticatory stress.

The energy needed to fracture a denture base under an impact, like accidentally dropping a denture, is defined as the impact strength (89). As per the NMA results, CCA demonstrated a higher value for impact strength when compared with CCM and TDP. CCI also indicated a higher value for impact strength when compared to TDP. Pairwise results indicate both CCI and CCA demonstrated significantly higher impact strength than CCM and TDP. However, the results are significantly impacted by values from one study.

Flexural modulus is the ability of a material to resist bending or breaking under stress (90). In the primary analysis, none of the interventions demonstrated significant results. However, sensitivity analysis excluding low quality studies indicate that CCI demonstrated significantly higher flexural modulus when compared with MIL, TDP and CCM. These results should be interpreted with caution, as the exclusion of low-quality studies that have included CCI as an intervention has caused the results to be significantly impacted by the values from one study.

Although the polymerization shrinkage of conventional acrylic-based denture bases is extensively documented and understood, material-related complications and failures of 3D-printed denture bases are significant and poorly understood (16). The printer-type, object topology, and post-print curing may overcome the initial printing accuracy of 3D-printed resins (91). It is interesting to note the significant number of fractures of denture bases in a clinical study, even though the manufacturer's instructions in fabrication and curing were thoroughly followed (92). The failure has been attributed to malalignment of the current ISO standards for newly introduced digitally fabricated denture base materials in comparison with conventional materials (53).

Structural flaws facilitate crack propagation and debonding during testing, contributing to a combination of adhesive and cohesive failure mechanisms in conventional and additively manufactured denture base resins. In a recent study comparing the physical properties of traditional teeth attached to a heat-cured denture base material compared to additively manufactured tooth-coloured materials attached to denture base-coloured materials,96% of both groups met the ISO 19736 standard for adhesive failure (<33%). The conventional group showed 5% adhesive and 95% mixed failure after thermocycling, likely due to voids at the bonding site caused by air entrapment or residual monomers. The additively manufactured group experienced 20% cohesive failure within the denture base resin, attributed to the strong interfacial adhesion resulting from its unified fabrication method. The remaining 80% showed mixed failure, possibly from porosities between printed layers and micropores formed under load (93). Porosity within the denture base may significantly influence the mechanical behaviour of the resin, including bond strength (94). Alternative techniques, such as microwave curing, may present challenges in achieving uniform polymerization, despite providing advantages in quicker curing times (95). Porosities in denture bases may further decrease the strength of the resin, leading to fracture. Subsequently, the long-term performance of the denture base may be impacted by the accumulation of debris, including plaque and calculus (96). To mitigate the impact of reduced strength, rubber-based polymers may be incorporated to enhance impact strength. This results in enhanced ability to absorb energy and fortify the resin (73).

The printing orientation of 3D-printed denture bases plays a crucial role in determining their mechanical properties. Optimal orientation influences strength, accuracy, time, and material wastage. A 45° printing orientation has improved accuracy and structural stability (97). A horizontal (0°) orientation offers the highest flexural strength. A vertical (90°) orientation provides improved flexural strength and microhardness (98). Enhanced surface quality and smoothness may result from orientations less than 45° by reducing support structures and minimal post-processing (99). The printing orientation affects both material consumption and printing time. Increased material use and time is associated with a vertical (90°) orientations as compared to horizontal orientations. Printing orientation for denture bases influences the properties and should be aligned to the requirements of the prosthesis and printer capabilities.

Milled denture bases offer several advantages due to the strong bonding between the teeth and the denture base. These include faster production, improved material properties such as reduced roughness and porosity, increased flexural and impact strength, enhanced hardness, better retention, and the convenience of quick replacement using patients' stored digital records. However, drawbacks include increased tooth wear and challenges maintaining the occlusal vertical dimension. Alternatively, technologies that allow separate fabrication of denture base resins (DBRs) and prefabricated teeth enable using materials with superior physico-chemical properties. Despite the benefits, CAD-CAM milling has notable downsides, such as significant material waste and wear of milling burs. This has led to growing interest in 3D printing as a more cost-effective alternative. However, current limitations of 3D-printed dentures—such as lower mechanical strength, inferior optical and aesthetic qualities, and reduced retention—make them less appealing. Continued development in 3D printing technology is needed to overcome these challenges and provide a viable alternative to traditional and milled dentures (100–103).

The authors acknowledge that frequentist network meta-analysis involves statistical assumptions, such as underlying distributions and variability across studies, which are standard in clinical evidence synthesis. The authors also recognize that these assumptions may not fully align with the characteristics of *in vitro* studies, which are often more deterministic and conducted under highly controlled conditions. The intent of this study is not to infer clinical probabilities or predictive outcomes, but rather to apply NMA as a comparative synthesis tool to examine relative intervention effects across the available *in vitro* evidence. Confidence intervals and ranking probabilities should be interpreted cautiously in this context, and our findings are exploratory, mechanistic, and not directly translatable to clinical decision-making.

The clinical impact of these findings is delineated below. A recent systematic review of clinical outcomes shows that digital dentures have comparable clinical properties to conventional ones (104). 3D printed dentures have demonstrated superior tissue adaptation and force distribution compared to traditional dentures (105, 106). Superior precision in fit has been observed for digitally fabricated dentures compared to conventional dentures. However, clinical efficiency for different dentures does not seem to have significant differences (107). Digitally fabricated dentures have also been found to be more time-efficient, requiring fewer appointments, chairside and lab time (108).

The fabrication costs of 3D-printed dentures have been reported to be between 200 and 400 USD. The overall fabrication costs of conventional dentures may seem lower when compared to digitally fabricated dentures; however, despite high initial costs, milled dentures may result in long-term savings due to reduced maintenance requirements. Conventional dentures may require the most time for fabrication, considering multiple appointments, try-ins, lab work, and adjustments. Printing for 3D-printed dentures can be completed in 1–2 h; however, the fabrication procedure involves processing and curing times that increase the overall time required (8, 109). Milled dentures can be fabricated more quickly than conventional dentures but may require more time than 3D-printed dentures (101, 110–112).

This section summarizes the limitations of the current review. Insufficient data was identified to provide meaningful conclusions regarding a few outcomes. Bias incorporated from higher values from one study diminishes the quality of evidence for impact strength. More trials comparing the impact strength between conventional and digital dentures are required to study this property further. Most of the included studies have not been performed and reported sufficiently on essential parameters like sample size and standardization of materials. Higher-quality studies are needed to establish recommendations.

## 5 Conclusion

Based on high-quality evidence, milled digital denture bases exhibit superior flexural strength to 3D-printed and conventionally fabricated denture bases under laboratory conditions. The data on superior mechanical properties must be validated clinically by high-quality, randomized, controlled clinical trials. Higher-quality studies are required to summarize the evidence for the remaining properties. Studies with a higher sample size and standardized protocols are needed to generate high-quality evidence.

## Data Availability

The original contributions presented in the study are included in the article/Supplementary Material, further inquiries can be directed to the corresponding author.
