# Supplementary material for: CAD-CAM vs. conventional denture bases: a systematic review with network meta-analysis of in vitro studies comparing strength, hardness, toughness, and elastic properties
Source: Front Dent Med. 2025 Aug 11;6:1638794. doi: 10.3389/fdmed.2025.1638794 (PMC12375453; doi:10.3389/fdmed.2025.1638794)
Supplement: Supplementary file 1 [file Datasheet1.docx]

**SUPPLEMENTARY TABLES**

**Supplementary Table 1.** PRISMA Abstract Checklist

| **Section and Topic** | **Item #** | **Checklist item** | **Reported (Yes/No)** |
| --- | --- | --- | --- |
| **TITLE** | | |  |
| Title | 1 | Identify the report as a systematic review. | Yes |
| **BACKGROUND** | | |  |
| Objectives | 2 | Provide an explicit statement of the main objective(s) or question(s) the review addresses. | Yes |
| **METHODS** | | |  |
| Eligibility criteria | 3 | Specify the inclusion and exclusion criteria for the review. | Yes |
| Information sources | 4 | Specify the information sources (e.g. databases, registers) used to identify studies and the date when each was last searched. | Yes |
| Risk of bias | 5 | Specify the methods used to assess risk of bias in the included studies. | Yes |
| Synthesis of results | 6 | Specify the methods used to present and synthesise results. | Yes |
| **RESULTS** | | |  |
| Included studies | 7 | Give the total number of included studies and participants and summarise relevant characteristics of studies. | Yes |
| Synthesis of results | 8 | Present results for main outcomes, preferably indicating the number of included studies and participants for each. If meta-analysis was done, report the summary estimate and confidence/credible interval. If comparing groups, indicate the direction of the effect (i.e. which group is favoured). | Yes |
| **DISCUSSION** | | |  |
| Limitations of evidence | 9 | Provide a brief summary of the limitations of the evidence included in the review (e.g. study risk of bias, inconsistency and imprecision). | Yes |
| Interpretation | 10 | Provide a general interpretation of the results and important implications. | Yes |
| **OTHER** | | |  |
| Funding | 11 | Specify the primary source of funding for the review. | No |
| Registration | 12 | Provide the register name and registration number. | Yes |

**Supplementary Table 2.** PRISMA Checklist

| **Section and Topic** | **Item #** | **Checklist item** | **Location where item is reported** |
| --- | --- | --- | --- |
| **TITLE** | | |  |
| Title | 1 | Identify the report as a systematic review. | Page 1 |
| **ABSTRACT** | | |  |
| Abstract | 2 | See the PRISMA 2020 for Abstracts checklist. | Page 1-2 |
| **INTRODUCTION** | | |  |
| Rationale | 3 | Describe the rationale for the review in the context of existing knowledge. | Page 3 (Abs) |
| Objectives | 4 | Provide an explicit statement of the objective(s) or question(s) the review addresses. | Page 3 (Abs) |
| **METHODS** | | |  |
| Eligibility criteria | 5 | Specify the inclusion and exclusion criteria for the review and how studies were grouped for the syntheses. | Page 5-6 (Material and Methods) |
| Information sources | 6 | Specify all databases, registers, websites, organisations, reference lists and other sources searched or consulted to identify studies. Specify the date when each source was last searched or consulted. | Page 5 |
| Search strategy | 7 | Present the full search strategies for all databases, registers and websites, including any filters and limits used. | Page 5 |
| Selection process | 8 | Specify the methods used to decide whether a study met the inclusion criteria of the review, including how many reviewers screened each record and each report retrieved, whether they worked independently, and if applicable, details of automation tools used in the process. | Page 5 |
| Data collection process | 9 | Specify the methods used to collect data from reports, including how many reviewers collected data from each report, whether they worked independently, any processes for obtaining or confirming data from study investigators, and if applicable, details of automation tools used in the process. | Page 5 |
| Data items | 10a | List and define all outcomes for which data were sought. Specify whether all results that were compatible with each outcome domain in each study were sought (e.g. for all measures, time points, analyses), and if not, the methods used to decide which results to collect. | Page 5 |
|  | 10b | List and define all other variables for which data were sought (e.g. participant and intervention characteristics, funding sources). Describe any assumptions made about any missing or unclear information. | Page 5 |
| Study risk of bias assessment | 11 | Specify the methods used to assess risk of bias in the included studies, including details of the tool(s) used, how many reviewers assessed each study and whether they worked independently, and if applicable, details of automation tools used in the process. | Page 5, 6  Supplementary Table 5 |
| Effect measures | 12 | Specify for each outcome the effect measure(s) (e.g. risk ratio, mean difference) used in the synthesis or presentation of results. | Page 5 |
| Synthesis methods | 13a | Describe the processes used to decide which studies were eligible for each synthesis (e.g. tabulating the study intervention characteristics and comparing against the planned groups for each synthesis (item #5)). | Page 5-6 |
|  | 13b | Describe any methods required to prepare the data for presentation or synthesis, such as handling of missing summary statistics, or data conversions. | Page 5-6 |
|  | 13c | Describe any methods used to tabulate or visually display results of individual studies and syntheses. | Page 5-6 |
|  | 13d | Describe any methods used to synthesize results and provide a rationale for the choice(s). If meta-analysis was performed, describe the model(s), method(s) to identify the presence and extent of statistical heterogeneity, and software package(s) used. | Page 5-6 |
|  | 13e | Describe any methods used to explore possible causes of heterogeneity among study results (e.g. subgroup analysis, meta-regression). | Page 6 |
|  | 13f | Describe any sensitivity analyses conducted to assess robustness of the synthesized results. | Page 6 |
| Reporting bias assessment | 14 | Describe any methods used to assess risk of bias due to missing results in a synthesis (arising from reporting biases). | Page 6 |
| Certainty assessment | 15 | Describe any methods used to assess certainty (or confidence) in the body of evidence for an outcome. | Page 6 |
| **RESULTS** | | |  |
| Study selection | 16a | Describe the results of the search and selection process, from the number of records identified in the search to the number of studies included in the review, ideally using a flow diagram. | Page 7, Figure 1 |
|  | 16b | Cite studies that might appear to meet the inclusion criteria, but which were excluded, and explain why they were excluded. | Page 27 |
| Study characteristics | 17 | Cite each included study and present its characteristics. | Page 7, Supplementary Table 4 |
| Risk of bias in studies | 18 | Present assessments of risk of bias for each included study. | Supplementary Table 5 |
| Results of individual studies | 19 | For all outcomes, present, for each study: (a) summary statistics for each group (where appropriate) and (b) an effect estimate and its precision (e.g. confidence/credible interval), ideally using structured tables or plots. | Page 6, page 19-22 |
| Results of syntheses | 20a | For each synthesis, briefly summarise the characteristics and risk of bias among contributing studies. | Supplementary Table 5 |
|  | 20b | Present results of all statistical syntheses conducted. If meta-analysis was done, present for each the summary estimate and its precision (e.g. confidence/credible interval) and measures of statistical heterogeneity. If comparing groups, describe the direction of the effect. | Page 7-10 |
|  | 20c | Present results of all investigations of possible causes of heterogeneity among study results. | Page 7-10 |
|  | 20d | Present results of all sensitivity analyses conducted to assess the robustness of the synthesized results. | Page 7-10 |
| Reporting biases | 21 | Present assessments of risk of bias due to missing results (arising from reporting biases) for each synthesis assessed. | Page 7-10 |
| Certainty of evidence | 22 | Present assessments of certainty (or confidence) in the body of evidence for each outcome assessed. | Page 7-10 |
| **DISCUSSION** | | |  |
| Discussion | 23a | Provide a general interpretation of the results in the context of other evidence. | Page 10-14 |
|  | 23b | Discuss any limitations of the evidence included in the review. | Page 15 |
|  | 23c | Discuss any limitations of the review processes used. | Page 15 |
|  | 23d | Discuss implications of the results for practice, policy, and future research. | Page 15 |
| **OTHER INFORMATION** | | |  |
| Registration and protocol | 24a | Provide registration information for the review, including register name and registration number, or state that the review was not registered. | Page 1, 5 |
|  | 24b | Indicate where the review protocol can be accessed, or state that a protocol was not prepared. | Page 1, 5 |
|  | 24c | Describe and explain any amendments to information provided at registration or in the protocol. | NA |
| Support | 25 | Describe sources of financial or non-financial support for the review, and the role of the funders or sponsors in the review. | Page 15 |
| Competing interests | 26 | Declare any competing interests of review authors. | Page 15 |
| Availability of data, code and other materials | 27 | Report which of the following are publicly available and where they can be found: template data collection forms; data extracted from included studies; data used for all analyses; analytic code; any other materials used in the review. | Supplementary material |

**Supplementary Table 3.** Search strategy

| Database | Search Terms |
| --- | --- |
| Pubmed | ((complete denture) OR (denture) OR (overlay denture)) OR (edentulism) OR (edentulous jaw)) AND ((computer aided design) OR (three dimensional printing) OR (CAD CAM denture) OR (CAD CAM) OR (computer aided manufacturing denture) OR (digital denture) OR (3D printed denture) OR (Stereolithography) OR (additive manufacturing) OR (milling) OR (material jetting) OR (digital light processing) OR (injection molding)) AND ((biomechanical phenomena) OR (elastic modulus) OR (shear strength) OR (wear resistance) OR (fracture resistance) OR (materials testing) OR (mechanical phenomena)) |
| Scopus | ( ( complete AND denture ) ) AND ( ( ( computer-aided AND design ) OR ( three AND dimensional AND printing ) OR ( cad AND cam AND denture ) OR ( computer AND aided AND manufacturing AND denture ) OR ( digital AND denture ) OR ( 3d AND printed AND denture ) ) OR ( ( stereolithography ) OR ( additive AND manufacturing ) OR ( milling ) OR ( material AND jetting ) OR ( digital AND light AND processing ) OR ( injection AND molding ) ) ) AND ( ( biomechanical AND phenomena ) OR ( elastic AND modulus ) OR ( shear AND strength ) OR ( wear AND resistance ) OR ( fracture AND resistance ) OR ( materials AND testing ) OR ( mechanical AND phenomena ) ) |
| Medline | ((complete denture or denture* or overlay denture or edentulism or edentulous jaw or resin base) and (((((((((computed aided design or three dimensional print* or CAD-CAM) adj2 denture) or CAD-CAM or computer aided manufactur*) adj2 denture) or digital) adj2 denture) or 3D print*) adj3 denture) or 3D print* or Stereolithography or additive manufactur* or milling or material jetting or digital light processing or injection molding) and (biomechanical phenomena or elastic modulus or shear strength or wear resistance or fracture resistance or materials test* or mechanical phenomena or flexural strength or hardness or surface roughness or microhardness)).mp. [mp=tx, bt, ti, ab, ct, ot, nm, hw, fx, kf, ox, px, rx, ui, sy, ux, mx] |

**Supplementary Table 4.** Characteristics of included in-vitro comparison studies.

| **Author (Year)** | **Outcome: Flexural strength (Mpa)** | | **Outcome: Hardness (VHN)** | | **Outcome: Impact Strength (kJ/m2)** | | **Outcome: Elastic modulus (MPa)** | | **Outcome: Fracture toughness (MPa·m1/2)** | | **Outcome: Flexural modulus (MPa)** | | **Outcome: Yield point (MPa)** | | **Outcome: Strain at yield point (MPa)** | | **Outcome: Toughness (N.mm)** | |
| --- | --- | --- | --- | --- | --- | --- | --- | --- | --- | --- | --- | --- | --- | --- | --- | --- | --- | --- |
|  | **Control** | **Experimental** | **Control** | **Experimental** | **Control** | **Experimental** | **Control** | **Experimental** | **Control** | **Experimental** | **Control** | **Experimental** | **Control** | **Experimental** | **Control** | **Experimental** | **Control** | **Experimental** |
| Prpić et al (2020) | CCM (PBH): NS  n=10 CCM (PAL):  NS n=10  CCM (IAH): NS  n=10 CCI: NS n=10 | MIL (IVC): NS n=10  MIL (IDP): NS n=10  MIL (POL): NS n=10  TDP: NS n=10 | CCM (PBH): NS  n=10 CCM (PAL):  NS n=10  CCM (IAH): NS  n=10 CCI: NS n=10 | MIL (IVC): NS n=10  MIL (IDP): NS n=10  MIL (POL): NS n=10  TDP: NS n=10 | Nil | Nil | Nil | Nil | Nil | Nil | Nil | Nil | Nil | Nil | Nil | Nil | Nil | Nil |
| Becerra et al (2021) | CCM: 73.6 ±  11.9 n=30  CCI: 78.2 ±  11.1 n=30 | MIL: 93.1 ±  3.4 n=30 | CCM: 23.9 ±  2.1 n=30  CCI: 23.1 ±  1.9 n=30 | MIL: 18.7 ±  1.7 n=30 | Nil | Nil | CCM: 2990 ±  13 n=30  CCI: 3320 ±  230 n=30 | MIL: 2600 ±  110 n=30 | Nil | Nil | Nil | Nil | Nil | Nil | Nil | Nil | Nil | Nil |
| Iwaki et al (2020) | CCM: 111.40  ± 7.30  n=5 | MIL: 124.08 ±  5.16  n=5 | Nil | Nil | Nil | Nil | Nil | Nil | Nil | Nil | CCM: 3660 ±  50  n=5 | MIL: 3790 ±  30  n=5 | Nil | Nil | Nil | Nil | Nil | Nil |
| Ayman et al (2017) | CCM: 62.38 ±  1.73 n=10 | MIL: 34.05 ±  2.32 n=10 | CCM: 1.348 ±  0.0897 n=10 | MIL: 2.285 ±  0.153 n=10 | Nil | Nil | Nil | Nil | Nil | Nil | Nil | Nil | Nil | Nil | Nil | Nil | Nil | Nil |
| Al-Dwairi et al (2020) | CCM: 93.33 ±  8.64 n=15 | MIL (SCH): 130.67 ±10.48 n=15  MIL (AD): 123.11 ±9.47 n=15 | Nil | Nil | CCM:14.756 ±2.106 n=15 | MIL (SCH) 29.56 ±6.94 n=15  MIL (AD) 24.556 ±2.633 n=15 | Nil | Nil | Nil | Nil | CCM: 2117.2 ±154.3 n=15 | MIL (SCH) 2474.7 ±249 n=15  MIL (AD) 2519.6 ±245.5 n=15 | Nil | Nil | Nil | Nil | Nil | Nil |
| †Al-Dwairi et al  (2019) | Nil | Nil | CCM: 18.09 ±  0.31 n=15 | MIL (AD): 20.60 ± 0.33 n=10  MIL (SCH): 19.80 ± 1.08 n=10 | Nil | Nil | Nil | Nil | Nil | Nil | Nil | Nil | Nil | Nil | Nil | Nil | Nil | Nil |
| Perea-Lowery et al (2021) | CCM: NS  n=8 CCA: NS  n=8 | MIL (IVC): NS  n=8  MIL (LT): NS  n=8  MIL (TB): NS  n=8 | CCM: NS  n=8 CCA: NS  n=8 | MIL (IVC): NS  n=8  MIL (LT): NS  n=8  MIL (TB): NS  n=8 | Nil | Nil | CCM: NS  n=8 CCA: NS  n=8 | MIL (IVC): NS  n=8  MIL (LT): NS  n=8  MIL (TB): NS  n=8 | Nil | Nil | Nil | Nil | Nil | Nil | Nil | Nil | Nil | Nil |
| Srinivasan et al  (2018) | CCM: 96±4  n=5 | MIL: 121±2  n=5 | CCM: 23.66 ±  1.53  n=2 | MIL: 22.53 ±  1.428  n=2 | Nil | Nil | CCM: 3900 ±  200  n=2 | MIL:4100 ± 200  n=2 | Nil | Nil | CCM: 2700±100  n=5 | MIL: 2700±200  n=5 | CCM: 54 ± 11  n=5 | MIL: 71 ± 6  n=5 | CCM: 0.020 ±  0.005  n=5 | MIL: 0.003 ±  0.002  n=5 | CCM: 436 ±46  n=5 | MIL: 956 ±85  n=5 |
| Aguirre et al (2020) | CCM: 116.6 ±  3.1 n=10  CCI: 86.7 ± 7.1 n=10 | MIL: 146.6 ±6.6 n=10 | Nil | Nil | Nil | Nil | Nil | Nil | Nil | Nil | CCM: 2918.4± 106.3 n=10  CCI: 2121.3 ±  176.6  n=10 | MIL: 3816.7 ±  44.3 n=10 | Nil | Nil | Nil | Nil | Nil | Nil |
| Steinmassl et al  (2018) | Nil | Nil | Nil | Nil | Nil | Nil | CCM: 3570.24  ± 450.75 n=10  CCA: 3405.01  ± 178.52 n=10 | MIL (WDD): 4009.95 ±  200.00 n=10  MIL (WNC): 4777.01 ±  110.72 n=10  MIL (WN): 4921.05 ±  87.85 n=10  MIL (AD): 4649.15 ±  1110.93 n=10  MIL (BDS): 4606.38 ±  325.93 n=10  MIL (VV): 4569.16 ±  267.40 n=10 | CCM: 1.25 ± 0.11  n=10  CCA: 1.11 ±0.08  n=10 | MIL (WDD): 1.73 ± 0.19 n=10  MIL (WNC): 1.31 ± 0.09 n=10  MIL (WN): 1.29 ± 0.6 n=10  MIL (AD): 1.04 ± 0.10 n=10  MIL (BDS): 1.02 ± 0.07 n=10  MIL (VV): 0.80 ± 0.07 n=10 | Nil | Nil | Nil | Nil | Nil | Nil | Nil | Nil |
| Alharethi et al (2024) | Nil | Nil | Nil | Nil | Nil | Nil | Nil | Nil | Nil | Nil | Nil | TDP (120°):  577.16 ± 26.54 n=22  TDP (135°):  687.00 ± 32.31 n=22  MIL: 1109.61  ± 57.91 n=22 | Nil | Nil | Nil | Nil | Nil | Nil |
| Casucci et al (2023) | CCM (R): 89.15 ± 14.31 n=10  CCM (P): 86.07 ± 7.09 n=10  CCM (F): 74.83 ± 7.84 n=10  CCM (HI): 85.58 ± 8.60 n=10  CCM (LL): 92.39 ± 17.18 n=10 CCM (FAST): 98.86 ± 10.66 n=10 | MIL (IVO): 91.88 ± 4.43 n=10  MIL (AAD): 107.87 ± 7.56 n=10  TDP (ND): 83.32 ± 8.38 n=10  TDP (GC): 96.87 ± 6.27 n=10  TDP (GCP): 102.96 ± 9.37 n=10  TDP (SPR): 85.44 ± 5.30 n=10 | Nil | Nil | Nil | Nil | Nil | Nil | Nil | Nil | Nil | Nil | Nil | Nil | Nil | Nil | Nil | Nil |
| Falahchai et al (2023) | CCM: 93.43 ±  3.55 n=17 | TDP: 67.95 ±  2.24 n=17 | CCM: 20.25 ±  2.01 n=17 | TDP: 14.14 ±  1.17 n=17 | CCM: 12.37 ±  2.59 n=17 | TDP: 18.29 ±  2.82 n=17 | Nil | Nil | Nil | Nil | CCM: 2123.28  ± 172.34 n=17 | TDP: 1791.35  ± 86.30  n=17 | Nil | Nil | Nil | Nil | Nil | Nil |
| †Srinivasan et al (2021) | Nil | MIL (AD):  114.108 ± 3.03 n=5  MIL (AVT):  114.508 ± 4.63 n=5  TDP (NCB): 99.684 ± 1.61  n=5 TDP (PB1):  90.756 ±  16.29  n=5 TDP (PB2):  67.348 ± 11.39 n=5  TDP (PB2V):  71.512 ± 10.77 n=5 | Nil | MIL (AD): 15.940 ± 0.36  n=5 MIL (AVT): 18.440 ± 0.99  n=5 TDP (NCB): 18.540 ± 1.31  n=5 TDP (PB1): 17.000 ± 1.26  n=5 TDP (PB2): 6.680 ± 2.26  n=5 | Nil | Nil | Nil | MIL (AD):3038 ± 80  n=5 MIL  (AVT):3064 ± 50  n=5 TDP  (NCB):2624 ± 40  n=5 TDP  (PB1):2716 ± 140  n=5 TDP  (PB2):2108 ± 40  n=5 TDP  (PB2V):1832  ± 220  n=5 | Nil | Nil | Nil | Nil | Nil | MIL (AD):8.134 ± 3.05  n=5 MIL  (AVT):5.538 ± 0.87  n=5 TDP  (NCB):5.658 ± 1.21  n=5 TDP  (PB1):5.818 ± 1.73  n=5 TDP  (PB2):4.346 ± 0.11  n=5 TDP  (PB2V):4.16 ± 0.07  n=5 | Nil | MIL (AD): 0.271 ± 0.11  n=5 MIL (AVT): 0.175 ± 0.03  n=5 TDP (NCB): 0.205 ± 0.05  n=5 TDP (PB1): 0.212 ± 0.06  n=5 TDP (PB2): 0.203 ± 0.01  n=5 TDP (PB2V): 0.211 ± 0.06  n=5 | Nil | MIL (AD):  794.322 ± 65.17 n=5  MIL (AVT): 678.984  ±137.27  n=5 TDP (NCB):  586.086 ± 105.69  n=5 TDP (PB1):  408.038 ± 262.94  n=5 TDP (PB2):  271.334 ± 192.55  n=5 TDP (PB2V):  414.050 ± 161.85  n=5 |
| Al-Qarni et al (2022) | CCM: 93.4  ±10.8 n=10 | TDP (ND): 56.4±4.7 n=10  TDP (ASG): NS n=10  TDP (FL2) : NS n=10 | Nil | Nil | Nil | Nil | CCM: NS n=10 | TDP (ND): NS n=10  TDP (ASG): NS n=10  TDP (FL2): NS n=10 | Nil | Nil | Nil | Nil | Nil | Nil | Nil | Nil | Nil | Nil |
| Çakmak et al (2023) | Nil | TDP: 56.74±14.16 n=20  MIL (IVO): 83.56 ±6.15 n=20  MIL (GC): 92.77 ±8.95 n=20 | Nil | TDP: 17.75  ±1.74 n=20  MIL (IVO): 18.96 ±1.28 n=20  MIL (GC): 25.35 ±1.13 n=20 | Nil | Nil | Nil | Nil | Nil | Nil | Nil | Nil | Nil | Nil | Nil | Nil | Nil | Nil |
| Kirad et al (2020) | CCM: 74.70±5.02 n=15  CCI: 84.82±5.30 n=15 | MIL: 93.16  ±5.46 n=15 | Nil | Nil | CCM: 8.446  ±0.937 n=15  CCI: 12.684  ±2.924 n=15 | MIL: 15.625  ±2.521 n=15 | Nil | Nil | Nil | Nil | Nil | Nil | Nil | Nil | Nil | Nil | Nil | Nil |
| Hada et al (2021) | CCA:95.1±4.3 n=10  CCM: 87.9±5 n=10 | MIL: 105.1±2.2 n=10 | Nil | Nil | Nil | Nil | Nil | Nil | Nil | Nil | CCA: 3000±100 n=10  CCM: 2800±0 n=10 | MIL: 2800±100 n=10 | Nil | Nil | Nil | Nil | Nil | Nil |
| †Perea-Lowery et al (2021) | CCM: NS n=16 CCA: NS n=16 | TDP (IPM): NS n=16  TDP (FL): NS n=16 | Nil | Nil | Nil | Nil | CCM: NS n=16 CCA: NS n=16 | TDP (IPM): NS n=16  TDP (FL): NS n=16 | CCM: NS n=12 CCA: NS n=12 | TDP (IPM): NS n=12  TDP (FL): NS n=12 | Nil | Nil | Nil | Nil | Nil | Nil | Nil | Nil |
| Chhabra et al (2022) | CCM: 92.01 ±  12.14 n=15 | TDP: 69.78 ±  7.54 n=15 | Nil | Nil | CCM:1.67 ± 0.79 n=15 | TDP:1.15 ± 0.40 n=15 | Nil | Nil | Nil | Nil | Nil | Nil | Nil | Nil | Nil | Nil | Nil | Nil |
| Fiore et al (2022) | CCM: 80.79±7.64  n=6 | MIL: 110.23±5.03  n=6 TDP:  87.34±6.39  n=6 | Nil | Nil | Nil | Nil | Nil | Nil | Nil | Nil | CCM: 2542.47± 301.55  n=6 | MIL: 3435.07± 346.34 n=6 TDP:  2371.37± 197.30  n=6 | Nil | Nil |  | Nil | Nil | Nil |
| Gad et al (2022) | CCM: 86.50 ±  0.98 n=10 | TDP: 69.02 ±  0.828 n=10  TDP (0.25%):  89.33 ± 3.688 n=10  TDP (0.50%):  79.88 ± 3.41 n=10 | CCM: 41.61 ±  2.16 n=10 | TDP: 34.53 ±  2.24 n=10  TDP (0.25%):  62.69 ± 2.34 n=10  TDP (0.50%):  63.33 ± 2.21 n=10 | CCM: 17.24 ±  0.93 n=10 | TDP: 9.86 ±  1.52 n=10  TDP (0.25%):10.81  ± 0.87 n=10  TDP (0.50%):12.09  ± 0.71 n=10 | Nil | Nil | Nil | Nil | Nil | Nil | Nil | Nil |  | Nil | Nil | Nil |
| †Gad et al (2022) | CCM: 86.63 ±  1.0 n=10 | TDP: 69.15  ±0.88 n=10 | CCM: 41.63  ±2.03 n=10 | TDP: 34.62  ±2.1 n=10 | CCM: 6.32 ±  0.50 n=10 | TDP: 2.44  ±0.31 n=10 | Nil | Nil | Nil | Nil | Nil | Nil | Nil | Nil |  | Nil | Nil | Nil |
| Helal et al (2022) | Nil | Nil | CCI: 14.39±0.89 n=30 | MIL: 26.14±0.39 n=30  TDP: 20.06±0.39  n=30 | CCI: 30.30±0.83  n=30 | MIL:25.88± 0.54 n=30  TDP:23.18± 0.71 n=30 | Nil | Nil | Nil | Nil | Nil | Nil | Nil | Nil |  | Nil | Nil | Nil |
| Lee et al (2022) | Nil | Nil | Nil | Nil | CCA: 14.9±  0.4 n=25  CCM: 8.9± 0.3 n=25 | TDP: 11.2±  0.7 n=25 | Nil | Nil | Nil | Nil | Nil | Nil | Nil | Nil | Nil |  | Nil | Nil |
| Mann et al (2022) | Nil | Nil | Nil | Nil | Nil | Nil | Nil | Nil | CCM: 2.09 ±  0.13 n=20 | MIL:1.96 ± 0.14 n=20  TDP: 2.23 ±0.11 n=20 | Nil | Nil | Nil | Nil | Nil |  | Nil | Nil |
| Neves et al (2022) | CCM(PBH): 107.7±27.70  n=8 CCM(VCR): 101.8±29.08  n=8 | TDP (VPD): 123.8±18.33  n=8 TDP (ND): 114.5±10.17  n=8 | **Measured in Knoop Microhardnes s (KHN)**    CCM (PBH): 14.1±0.69  n=8  CCM (VCR): 14.9±0.41  n=8 | **Measured in Knoop Microhardnes s (KHN)**    TDP (VPD): 11.6±0.34  n=8  TDP (ND): 12.5±0.29  n=8 | Nil | Nil | Nil | Nil | Nil | Nil | Nil | Nil | Nil | Nil |  | Nil | Nil | Nil |
| Zeidan et al (2022) | Nil | Nil | CCM: 22.44±0.98 n=10 | MIL: 29.18 ±  3.44  n=10  TDP: 2.64 ±  0.37  n=10 | Nil | Nil | CCM: 3017.16  ± 215.32 n=10 | MIL: 3240.06  ± 61.23 n=10  TDP: 576.65 ±  37.73  n=10 | Nil | Nil | Nil | Nil | Nil | Nil |  | Nil | Nil | Nil |
| ††Al-Dwairi et al (2023) | CCM: 92.44  ±7.91  n=15 | TDP (DTN): 81.33±5.88  n=15  TDP (ASG): 79.33±6.07  n=15  TDP (ND): 74.89±8.44  n=15 | CCM: 18.11  ±0.65  n=15 | TDP (DTN): 16.41±0.96  n=15  TDP (ASG): 16.24 ±0.79  n=15  TDP (ND): 16.20 ±0.93 n=15 | **Unit in kg/m2**    CCM: 16.64  ±1.69 | **Unit in kg/m2**    TDP (DTN): 17.98 ±1.76 n=15  TDP (ASG):16.76  ±1.75 n=15  TDP (ND): 15.20 ±0.69  n=15 | Nil | Nil | Nil | Nil | CCM: 2084.99  ±180.33  n=15 | TDP (DTN): 2115.80  ±178.95 n=15  TDP (ASG): 1801.40  ±176.86 n=15  TDP (ND): 1685.09  ±157.14   n=15 | Nil | Nil |  | Nil | Nil | Nil |
| †Zeidan et al (2023) | CCM: 99.00±16.53 n=10  CCI: 33.28±0.79 n=10 | MIL (AD): 120.49 ±13.07 n=10  MIL (POL): 119.59 ±6.25 n=10  TDP (ND): 61.63 ±2.32 n=10  TDP (HAR): 28.82 ±1.94  n=10 | Nil | Nil | Nil | Nil | Nil | Nil | Nil | Nil | Nil | Nil | Nil | Nil | Nil | Nil | Nil | Nil |
| Greil et al (2023) | CCM:  66.32 ±  4.58  n=30 | MIL: 71.50 ±  5.79  n=30  TDP (ND): 90.44 ± 4.10 n=30  TDP (FOT): 69.75 ± 3.65 n=30  TDP (FP): 96.13 ± 8.41 n=30  TDP (VPD): 99.57 ± 4.89 n=30 | Nil | Nil | Nil | Nil | Nil | Nil | Nil | Nil | Nil | Nil | Nil | Nil | Nil | Nil | Nil | Nil |
| Freitas et al (2023) | CCM (LUCI): 108.94 ± 9.14 n=10  CCM (VPW):  108.09 ± 11.75 n=10 | MIL: 114.96 ±  16.23  n=10  TDP: 57.23 ±  9.07  n=10 | Nil | Nil | Nil | Nil | Nil | Nil | Nil | Nil | Nil | Nil | Nil | Nil | Nil | Nil | Nil | Nil |
| Fouda et al (2023) | CCM: 82.7 ±  1.60  n=10 | MIL(AD): 94.5 ± 2.20  n=10  MIL (IVC): 87.6 ± 1.10  n=10  TDP (ASG): 70.1 ± 0.80  n=10  TDP (FL2): 69.1 ± 1.5  n=10  TDP (ND): 67.7 ± 1.20  n=10 | CCM: 39.6 ±  9.90  n=10 | MIL(AD): 46.3 ± 2.90  n=10  MIL (IVC): 46.9 ± 5.90  n=10  TDP (ASG): 31.1 ± 7.50  n=10  TDP (FL2):17.50 ± 2.80  n=10  TDP (ND): 15.2 ± 0.15  n=10 | Nil | Nil | CCM: 6458.8  ± 296.6 n=10 | MIL(AD): 8547.5 ± 397.5  n=10  MIL (IVC): 9507.4 ±  479.1  n=10  TDP (ASG): 5258.9 ±  325.9  n=10  TDP (FL2): 4792.1 ±  421.6  n=10  TDP (ND): 4750.3 ±288.2  n=10 | Nil | Nil | Nil | Nil | Nil | Nil | Nil | Nil | Nil | Nil |
| Alhotan et al (2025) | CCM: 71.9 ±11.6  n=15 | MIL: 103.4 ±2.8  n=15  TDP: 86.7 ±8.3  n=15 | Nil | Nil | Nil | Nil | CCM: 2581.8 ±247.1  n=15 | MIL: 3127.0 ±131.1  n=15  TDP: 2505.8±175.6  n=15 | Nil | Nil | Nil | Nil | Nil | Nil | Nil | Nil | Nil | Nil |
| Cantelli et al (2024) | CCM: 52.8 ±3.5  n=30 | TDP: 31.9 ±8.8  n=30 | Nil | Nil | Nil | Nil | CCM: 1172 ±99  n=30 | TDP: 539 ±115  n=30 | Nil | Nil | Nil | Nil | Nil | Nil | Nil | Nil | Nil | Nil |
| Arora et al (2024) | CCM: 152.96 ± 27 n=6  CCI: 131.28 ± 22.21 n=6 | TDP: 112.7 ± 4.54 n=6  MIL: 130.08 ± 7.73 n=6 | CCM: 20.05 ± 1.13 n=6  CCI: 16.56 ± 0.90 n=6 | TDP: 13.76 ± 2.22 n=6  MIL: 14.60 ± 0.36 n=6 | Nil | Nil | Nil | Nil | Nil | Nil | Nil | Nil | Nil | Nil | Nil | Nil | Nil | Nil |
| Alshali et al (2024) | Nil | TDP (DTC): 128.55 ±10.30 n=12  TDP (FL): 122.52 ±13.66 n=12  TDP (LUC): 108.12 ±2.17 n=12  MIL (AD): 146.00 ±12.53 n=12  MIL (LUCI): 125.51 ±14.84 n=12  MIL (KMI): 140.44 ±4.26 n=12 | Nil | Nil | Nil | Nil | Nil | Nil | Nil | Nil | Nil | TDP (DTC): 1150 ±270  n=12  TDP (FL): 1420 ±280  n=12  TDP (LUC): 1500 ±300  n=12  MIL (AD): 1880 ±380  n=12  MIL (LUCI): 2090 ±610  n=12  MIL (KMI): 1740 ±380  n=12 | Nil | Nil | Nil | Nil | Nil | Nil |
| Souza et al (2024) | CCM (Base): 75 ± 1.9 n=5  CCM (Tooth): 95 ± 11.7 n=5  CCI (Base): 69 ± 6.6 n=5  CCI (Tooth): 71 ± 8.3 n=5 | TDP: 77 ± 0.8 n=5  MIL: 99 ± 3.1 n=5 | Nil | Nil | Nil | Nil | Nil | Nil | Nil | Nil | Nil | Nil | Nil | Nil | Nil | Nil | Nil | Nil |
| Yu et al  (2024) | CCI: 95.28 ±5.59 n=10 | TDP: 119.11 ±9.29  n=10  MIL: 93.01 ±6.27  n=10 | Nil | Nil | Nil | Nil | Nil | Nil | Nil | Nil | CCI: 1420 ±200  n=10 | TDP: 2380 ±190 n=10  MIL: 1920 ±180 n=10 | Nil | Nil | Nil | Nil | Nil | Nil |
| Temizci et al (2024) | CCM: 94.23±10.40 n=10 | MIL: 104.65±5.12 n=10  TDP: 113.53±7.94 n=10 | Nil | Nil | Nil | Nil | Nil | Nil | Nil | Nil | Nil | Nil | Nil | Nil | Nil | Nil | Nil | Nil |
| Lawson et al (2024) | CCM: 96.36 ± 2.43  n=10 | MIL (IVO): 77.01 ± 1.72 n=10  TDP (DTC): 97.03 ± 4.87 n=10  TDP (SPR2): 102.40 ± 3.15 n=10  TDP (LUC): 82.36 ± 1.68 n=10  TDP (TDR): 101.76 ± 7.65 n=10 | Nil | Nil | Nil | Nil | Nil | Nil | CCM: 2.03 ± 0.12  n=10 | MIL (IVO): 1.87 ± 0.09  n=10  TDP (DTC): 0.54 ± 0.05  n=10  TDP (SPR2): 1.76 ± 0.15  n=10  TDP (LUC): 2.01 ± 0.09  n=10  TDP (TDR): 0.58 ± 0.05  n=10 | Nil | Nil | Nil | Nil | Nil | Nil | Nil | Nil |
| Arora et al (2024) | Nil | Nil | Nil | TDP (ASG): 13.76±2.22 n=6  MIL (IVO): 14.6±0.36 n=6  TDP (ASG2): 18.28±2.21 n=6  MIL (IVD): 22.26±1.96 n=6 | Nil | Nil | Nil | Nil | Nil | Nil | Nil | Nil | Nil | Nil | Nil | Nil | Nil | Nil |
| Vuksic et al (2024) | CCM: 97.06±6.25 n=5  CCI: 62.57±5.69 n=5 | MIL (IVC): 79.06±4.65  n=5  MIL (POL): 96.27±5.81  n=5  MIL (ANA): 83.31±3.21  n=5  TDP (FP): 103.33±16.71  n=5  TDP (IPM): 69.75±7.63  n=5 | CCM: 20.58±0.52 n=8  CCI: 10.61±0.24 n=8 | MIL (IVC): 17.23±0.99  n=8  MIL (POL): 22.86±0.72  n=8  MIL (ANA): 18.83±0.48  n=8  TDP (FP): 21.3±0.45  n=8  TDP (IPM): 16.55±0.81  n=8 | Nil | Nil | Nil | Nil | Nil | Nil | Nil | Nil | Nil | Nil | Nil | Nil | Nil | Nil |
| El Samahy et al (2023) |  | MIL: NS  n=7  TDP: NS  n=7 | Nil | Nil | Nil | MIL: NS  n=7  TDP: NS  n=7 | Nil | Nil | Nil | Nil | Nil | Nil | Nil | Nil | Nil | Nil | Nil | Nil |
| Patankar et al (2022) | CCM: 71.72±11.58 n=15  CCI: 84.42±10.42 n=15 | MIL: 97.46±9.93 n=15 | Nil | Nil | Nil | Nil | Nil | Nil | Nil | Nil | Nil | Nil | Nil | Nil | Nil | Nil | Nil | Nil |
| Pacquet et al (2019) | CCM: 97.31±4.96 n=25  CCI: 79.35±10.01 n=25 | MIL: 87.98±7.37 n=25 | CCM: 19.46±0.40 n=10  CCI: 16.85±0.44 n=10 | MIL: 19.31±1.48 n=10 | Nil | Nil | Nil | Nil | Nil | Nil | Nil | Nil | Nil | Nil | Nil | Nil | Nil | Nil |

Abbreviations: AAD=AADVA Disc; AD=AvaDent Digital Dentures; ASG=ASIGA DentaBASE; AVT=Avadent Extreme CADCAM shaded puck; BDS=Baltic Denture System; CCA=Conventional autopolymerisation; CCI=Conventional injection moulding; CCM=Conventional compression moulding; DTN=Dentona Optiprint Denture 3D Printer resin; F=Acryself; FAST=Acrypol FAST; FL=FormLabs Denture Base OP resin; FL2=FormLabs Denture Base LP resin; FOT=Fotodent Denture; FP=FREEPRINT denture (Detax); GC=GC Temp Print; GC=GCAM, Graphenano Dental SL; GCP=GC Temp Print + Pink; HAR=Harz Labs Dental Pink; HI=Acrypol HI; IAH=Interacryl Hot; IDP=Interdent CC disc PMMA; IPM=IMPRIMO® cure LC Denture Scheu-Dental GmbH; IVC=IvoBase CAD; IVO=Ivotion Base; LL=Acrypol LL; LT=Degos Dental L-Temp; LUCI=Lucitone 199; MIL=CAD-CAM milled; NCB=NextDent C&B; ND=NextDent Denture 3D+; P=Acryself P; PAL=Paladon 65; PB1=NextDent Base printed with recommended 3D-printer (Rapid Shape D30); PB2=NextDent Base printed with third-party 3D-printer; PB2V=NextDent Base printed with vertical orientation; PBH=Probase Hot; POL - PINK CAD-CAM DISC (Polident); R=Acrypol R; SCH=Schütz Dental; SPR=SprintRay EU Denture Base; TB=Zirkonzahn Temp Basic Tissue; TDP (0.25%)=TDP with 0.25% of incorporating silicon dioxide nanoparticles (SNPs); TDP (0.50%)=TDP with 0.50% of incorporating silicon dioxide nanoparticles (SNPs); TDP (120°)=3D printed at a 120-degree build orientation; TDP (135°)=3D printed at a 135-degree build orientation; TDP=Three-dimensional printed; VCR=Villacryl Rapid; VPD=V-Print Dentbase (VOCO); VPW=VipiWave; VV=Vita VIONIC; WDD=Wieland Digital Dentures; WN=Whole You Nexteeth; WNC=Whole You Nexteeth with light-curing topcoat.

**Supplementary Table 5.** RoBDEMAT assessment of included in-vitro comparison studies in meta-analyses

| Author | Y    Year | Domain 1: Bias in planning and allocation | | | Domain 2: Bias in sample/specimen preparation | | Domain 3: Bias in outcome assessment | | Domain 4: Bias in data treatment and outcome reporting | | Grade |
| --- | --- | --- | --- | --- | --- | --- | --- | --- | --- | --- | --- |
|  |  | 1.1: Control group | 1.2: Randomization of samples | 1.3: Sample size rationale and reporting | 2.1: Standardization of samples  and materials | 2.2: Identical experimental conditions across groups | 3.1: Adequate and standardized testing procedures and outcomes | 3.2: Blinding of the test operator | 4.1: Statistical analysis | 4.2: Reporting study outcomes |  |
| Becerra et al | 2021 | Sufficiently reported | Not applicable | Insufficiently reported | Sufficiently reported | Sufficiently reported | Adequate | Not applicable | Sufficiently reported | Sufficiently reported | Low |
| Iwaki et al | 2020 | Sufficiently reported | Not applicable | Not reported | Insufficiently reported | Sufficiently reported | Adequate | Not applicable | Sufficiently reported | Sufficiently reported | High |
| Ayman et al | 2017 | Sufficiently reported | Not applicable | Not reported | Insufficiently reported | Sufficiently reported | Adequate | Not applicable | Insufficiently reported | Sufficiently reported | High |
| Al-Dwairi et al | 2020 | Sufficiently reported | Not applicable | Not reported | Insufficiently reported | Sufficiently reported | Adequate | Not applicable | Sufficiently reported | Sufficiently reported | High |
| †Al-Dwairi et al | 2019 | Sufficiently reported | Not applicable | Not reported | Sufficiently reported | Sufficiently reported | Adequate | Not applicable | Sufficiently reported | Sufficiently reported | Low |
| Srinivasan et al | 2018 | Sufficiently reported | Not applicable | Not reported | Sufficiently reported | Sufficiently reported | Adequate | Not applicable | Sufficiently reported | Sufficiently reported | Low |
| Aguirre et al | 2020 | Sufficiently reported | Not applicable | Not reported | Insufficiently reported | Sufficiently reported | Adequate | Not applicable | Sufficiently reported | Sufficiently reported | High |
| Steinmassl et al | 2018 | Sufficiently reported | Not applicable | Not reported | Insufficiently reported | Sufficiently reported | Adequate | Not applicable | Sufficiently reported | Sufficiently reported | High |
| Alharethi et al | 2024 | Not applicable | Not applicable | Insufficiently reported | Sufficiently reported | Sufficiently reported | Adequate | Not applicable | Sufficiently reported | Sufficiently reported | Low |
| Casucci et al | 2023 | Sufficiently reported | Not applicable | Not reported | Sufficiently reported | Sufficiently reported | Adequate | Not applicable | Sufficiently reported | Sufficiently reported | Low |
| Falahchai et al | 2023 | Sufficiently reported | Not applicable | Insufficiently reported | Sufficiently reported | Sufficiently reported | Adequate | Not applicable | Sufficiently reported | Sufficiently reported | Low |
| †Srinivasan et al | 2021 | Not applicable | Not applicable | Not reported | Insufficiently reported | Sufficiently reported | Adequate | Not applicable | Sufficiently reported | Sufficiently reported | High |
| Al-Ǫarni et al | 2022 | Sufficiently reported | Not applicable | Not reported | Sufficiently reported | Sufficiently reported | Adequate | Not applicable | Sufficiently reported | Sufficiently reported | Low |
| Çakmak et al | 2023 | Not applicable | Not applicable | Insufficiently reported | Insufficiently reported | Sufficiently reported | Adequate | Not applicable | Sufficiently reported | Sufficiently reported | High |
| Kirad et al | 2020 | Sufficiently reported | Not applicable | Not reported | Sufficiently reported | Sufficiently reported | Adequate | Not applicable | Sufficiently reported | Sufficiently reported | Low |
| Hada et al | 2021 | Sufficiently reported | Not applicable | Not reported | Insufficiently reported | Insufficiently reported | Adequate | Not applicable | Sufficiently reported | Sufficiently reported | High |
| Chhabra et al | 2022 | Sufficiently reported | Not applicable | Not reported | Insufficiently reported | Insufficiently reported | Adequate | Not applicable | Sufficiently reported | Sufficiently reported | High |
| Fiore et al | 2022 | Sufficiently reported | Not applicable | Not reported | Sufficiently reported | Sufficiently reported | Adequate | Not applicable | Insufficiently reported | Sufficiently reported | High |
| Gad et al | 2022 | Sufficiently reported | Not applicable | Sufficiently reported | Sufficiently reported | Sufficiently reported | Adequate | Not applicable | Sufficiently reported | Sufficiently reported | Low |
| †Gad et al | 2022 | Sufficiently reported | Not applicable | Sufficiently reported | Sufficiently reported | Sufficiently reported | Adequate | Not applicable | Sufficiently reported | Sufficiently reported | Low |
| Helal et al | 2022 | Sufficiently reported | Not applicable | Not reported | Sufficiently reported | Sufficiently reported | Adequate | Not applicable | Sufficiently reported | Sufficiently reported | Low |
| Lee et al | 2022 | Sufficiently reported | Not applicable | Not reported | Sufficiently reported | Sufficiently reported | Adequate | Not applicable | Sufficiently reported | Sufficiently reported | Low |
| Mann et al | 2022 | Sufficiently reported | Not applicable | Sufficiently reported | Insufficiently reported | Sufficiently reported | Adequate | Not applicable | Insufficiently reported | Sufficiently reported | High |
| Neves et al | 2022 | Sufficiently reported | Not applicable | Sufficiently reported | Sufficiently reported | Sufficiently reported | Adequate | Not applicable | Sufficiently reported | Sufficiently reported | Low |
| Zeidan et al | 2022 | Sufficiently reported | Not applicable | Sufficiently reported | Sufficiently reported | Sufficiently reported | Adequate | Not applicable | Sufficiently reported | Sufficiently reported | Low |
| ††Al-Dwairi et al | 2023 | Sufficiently reported | Not applicable | Not reported | Sufficiently reported | Sufficiently reported | Adequate | Not applicable | Sufficiently reported | Sufficiently reported | Low |
| †Zeidan et al | 2023 | Sufficiently reported | Not applicable | Sufficiently reported | Insufficiently reported | Insufficiently reported | Adequate | Not applicable | Sufficiently reported | Sufficiently reported | High |
| Greil et al | 2023 | Sufficiently reported | Not applicable | Not reported | Sufficiently reported | Sufficiently reported | Adequate | Not applicable | Sufficiently reported | Sufficiently reported | Low |
| Freitas et al | 2023 | Sufficiently reported | Not applicable | Not reported | Insufficiently reported | Insufficiently reported | Adequate | Not applicable | Sufficiently reported | Sufficiently reported | High |
| Fouda et al | 2023 | Sufficiently reported | Not applicable | Sufficiently reported | Insufficiently reported | Sufficiently reported | Adequate | Not applicable | Insufficiently reported | Sufficiently reported | High |
| Alhotan et al | 2025 | Sufficiently reported | Not applicable | Sufficiently reported | Sufficiently reported | Sufficiently reported | Adequate | Not applicable | Sufficiently reported | Sufficiently reported | Low |
| Cantelli et al | 2024 | Sufficiently reported | Not applicable | Not reported | Sufficiently reported | Sufficiently reported | Adequate | Not applicable | Insufficiently reported | Sufficiently reported | High |
| Arora et al | 2024 | Sufficiently reported | Not applicable | Sufficiently reported | Insufficiently reported | Sufficiently reported | Adequate | Not applicable | Sufficiently reported | Sufficiently reported | Low |
| Alshali et al | 2024 | Not applicable | Not applicable | Not reported | Sufficiently reported | Insufficiently reported | Adequate | Not applicable | Sufficiently reported | Sufficiently reported | High |
| Souza et al | 2024 | Sufficiently reported | Not applicable | Sufficiently reported | Sufficiently reported | Sufficiently reported | Adequate | Not applicable | Sufficiently reported | Sufficiently reported | Low |
| Yu et al | 2024 | Sufficiently reported | Not applicable | Not reported | Insufficiently reported | Sufficiently reported | Adequate | Not applicable | Sufficiently reported | Sufficiently reported | High |
| Temizci et al | 2024 | Sufficiently reported | Not applicable | Sufficiently reported | Sufficiently reported | Sufficiently reported | Adequate | Not applicable | Sufficiently reported | Sufficiently reported | Low |
| Lawson et al | 2024 | Sufficiently reported | Not applicable | Insufficiently reported | Sufficiently reported | Sufficiently reported | Adequate | Not applicable | Sufficiently reported | Sufficiently reported | Low |
| Arora et al | 2024 | Not applicable | Not applicable | Not reported | Sufficiently reported | Sufficiently reported | Adequate | Not applicable | Sufficiently reported | Sufficiently reported | Low |
| Vuksic et al | 2024 | Sufficiently reported | Not applicable | Sufficiently reported | Insufficiently reported | Insufficiently reported | Adequate | Not applicable | Sufficiently reported | Sufficiently reported | High |
| Patankar et al | 2022 | Sufficiently reported | Not applicable | Not reported | Insufficiently reported | Sufficiently reported | Adequate | Not applicable | Insufficiently reported | Sufficiently reported | High |
| Pacquet et al | 2019 | Sufficiently reported | Not applicable | Not reported | Insufficiently reported | Insufficiently reported | Adequate | Not applicable | Insufficiently reported | Sufficiently reported | High |

**Supplementary Table 6.** Results of network meta-analysis: Flexural strength with SUCRA ranking.

| **Interventions** | **Flexural strength** | | |
| --- | --- | --- | --- |
|  | **SMD (95% CI)** | **P** | **SUCRA rank (score)** |
| **MIL** | 0.70 (-0.37, 1.77) | 0.201 | 1 (87.1) |
| **CCM** | Reference | | 2 (61.4) |
| **CCA** | 0.02 (-4.55, 4.59) | 0.992 | 3 (58.6) |
| **TDP** | -0.92 (-2.02, 0.18) | 0.102 | 4 (26.5) |
| **CCI** | -1.33 (-2.86, 0.21) | 0.09 | 5 (16.5) |

Abbreviations: CCM, conventional compression moulding; CCI, conventional injection moulding; CCA, conventional autopolymerisation; MIL, CAD-CAM milled; TDP, three-dimensional printed

**Supplementary Table 7.** League table showing the results of sensitivity analysis by excluding low quality studies: Flexural strength.

| **CCI** | NA | NA | NA |
| --- | --- | --- | --- |
| *-1.74 (-4.48,1.01)* | **MIL** | NA | NA |
| *0.39 (-2.44,3.22)* | *2.13 (0.21,4.05)** | **TDP** | NA |
| *-0.64 (-3.33,2.04)* | *1.09 (-0.73,2.91)* | *-1.04 (-2.70,0.62)* | **CCM** |

Note: * indicates P < 0.05, Network results are denoted in italics.

Abbreviations: CCI, Conventional injection moulding; MIL, CAD-CAM milled; TDP, Three-dimensional printed; CCM, Conventional compression moulding

**Supplementary Table 8.** Results of network meta-analysis: Hardness with SUCRA ranking.

| **Interventions** | **Hardness** | | |
| --- | --- | --- | --- |
|  | **SMD (95% CI)** | **P** | **SUCRA rank (score)** |
| **MIL** | 0.99 (-1.73, 3.72) | 0.475 | 1 (91.4) |
| **CCM** | Reference | | 2 (69.6) |
| **TDP** | -1.88 (-4.58, 0.83) | 0.174 | 3 (28.2) |
| **CCI** | -3.07 (-6.71, 0.57) | 0.098 | 4 (10.8) |

Abbreviations: CCM, conventional compression moulding; CCI, conventional injection moulding; MIL, CAD-CAM milled; TDP, three-dimensional printed

**Supplementary Table 9.** League table showing the results of sensitivity analysis by excluding low quality studies: Hardness.

| **CCI** | NA | NA | NA |
| --- | --- | --- | --- |
| *-5.43 (-11.36,0.51)* | **MIL** | NA | NA |
| *-1.34 (-7.42,4.74)* | *4.09 (-0.66,8.85)* | **TDP** | NA |
| *-4.26 (-10.33,1.81)* | *1.17 (-3.56,5.89)* | *-2.93 (-7.05,1.19)* | **CCM** |

Note: * indicates P < 0.05, Network results are denoted in italics.

Abbreviations: CCI, Conventional injection moulding; MIL, CAD-CAM milled; TDP, Three-dimensional printed; CCM, Conventional compression moulding

**Supplementary Table 10.** Results of network meta-analysis: Impact strength with SUCRA ranking.

| **Interventions** | **Impact strength** | | |
| --- | --- | --- | --- |
|  | **SMD (95% CI)** | **P** | **SUCRA rank (score)** |
| **CCA** | 8.88 (1.17, 16.59) | 0.024* | 1 (92.3) |
| **CCI** | 4.88 (-1.07, 10.83) | 0.108 | 2 (74.1) |
| **MIL** | 2.13 (-2.90, 7.16) | 0.407 | 3 (48.2) |
| **CCM** | Reference | | 4 (26.9) |
| **TDP** | -1.36 (-4.91, 2.19) | 0.452 | 5 (8.4) |

Note: presence of (*) and texts in red indicate intervention statistically significant (P<.05).

Abbreviations: CCM, conventional compression moulding; CCI, conventional injection moulding; CCA, conventional autopolymerisation; MIL, CAD-CAM milled; TDP, three-dimensional printed

**Supplementary Table 11.** League table showing the results of sensitivity analysis by excluding low quality studies: Impact strength.

| **CCA** | NA | NA | NA | NA |
| --- | --- | --- | --- | --- |
| *3.71 (-7.67,15.08)* | **CCI** | NA | NA | NA |
| *6.17 (-5.19,17.53)* | *2.46 (-4.66,9.59)* | **MIL** | NA | NA |
| *10.29 (1.15,19.44)** | *6.59 (-0.95,14.12)* | *4.13 (-3.38,11.63)* | **TDP** | NA |
| *8.89 (-0.28,18.06)* | *5.18 (-2.34,12.70)* | *2.72 (-4.78,10.22)* | *-1.41 (-6.12,3.30)* | **CCM** |

Note: * indicates P < 0.05, Network results are denoted in italics.

Abbreviations: CCA, Conventional autopolymerisation; CCI, Conventional injection moulding; MIL, CAD-CAM milled; TDP, Three-dimensional printed; CCM, Conventional compression moulding

**Supplementary Table 12.** Results of network meta-analysis: Elastic modulus with SUCRA ranking.

| **Interventions** | **Elastic modulus** | | |
| --- | --- | --- | --- |
|  | **SMD (95% CI)** | **P** | **SUCRA rank (score)** |
| **CCI** | 3.34 (-5.56, 12.24) | 0.462 | 1 (79.7) |
| **MIL** | 0.38 (-3.88, 4.64) | 0.860 | 2 (60.2) |
| **CCM** | Reference | | 3 (53.3) |
| **CCA** | -0.46 (-9.37, 8.44) | 0.919 | 4 (50.8) |
| **TDP** | -5.16 (-9.92, -0.39) | 0.034* | 5 (6.1) |

Note: presence of (*) and texts in red indicate intervention statistically significant (P<.05).

Abbreviations: CCM, conventional compression moulding; CCI, conventional injection moulding; CCA, conventional autopolymerisation; MIL, CAD-CAM milled; TDP, three-dimensional printed

**Supplementary Table 13.** League table showing the results of sensitivity analysis by excluding low quality studies: Elastic modulus.

| **CCI** | NA | NA | NA |
| --- | --- | --- | --- |
| *2.81 (-11.34,16.97)* | **MIL** | NA | NA |
| *12.90 (-3.76,29.56)* | *10.09 (-0.77,20.95)* | **TDP** | NA |
| *3.48 (-10.67,17.63)* | *0.67 (-8.28,9.63)* | *-9.42 (-20.23,1.40)* | **CCM** |

Note: * indicates P < 0.05, Network results are denoted in italics.

Abbreviations: CCI, Conventional injection moulding; MIL, CAD-CAM milled; TDP, Three-dimensional printed; CCM, Conventional compression moulding

**Supplementary Table 14.** Results of network meta-analysis: Flexural modulus with SUCRA ranking.

| **Interventions** | **Flexural modulus** | | |
| --- | --- | --- | --- |
|  | **SMD (95% CI)** | **P** | **SUCRA rank (score)** |
| **CCA** | 2.87 (-3.14, 8.88) | 0.349 | 1 (82.6) |
| **MIL** | 0.98 (-1.39, 3.35) | 0.419 | 2 (70.2) |
| **CCM** | Reference | | 3 (44.8) |
| **TDP** | -0.13 (-3.09, 2.82) | 0.930 | 4 (43.3) |
| **CCI** | -2.43 (-6.22, 1.36) | 0.209 | 5 (9.0) |

Abbreviations: CCM, conventional compression moulding; CCI, conventional injection moulding; CCA, conventional autopolymerisation; MIL, CAD-CAM milled; TDP, three-dimensional printed

**Supplementary Table 15**. League table showing the network and pairwise results for flexural modulus.

| **CCA** | NA | 2.00 (0.91, 3.09) * | NA | NA |
| --- | --- | --- | --- | --- |
| *5.30 (-1.58, 12.18)* | **CCI** | -3.51 (-10.02, 3.00) | -4.92 (-6.75, -3.09) * | -1.79 (-8.88, 5.30) |
| *1.89 (-4.12, 7.90)* | *-3.41 (-7.11, 0.30)* | **MIL** | 0.69 (-0.91, 2.29) | 1.89 (-0.50, 4.28) |
| *3.00 (-3.46, 9.46)* | *-2.30 (-6.45, 1.86)* | *1.11 (-1.74, 3.96)* | **TDP** | -1.13 (-2.51, 0.26) |
| *2.87 (-3.14, 8.88)* | *-2.43 (-6.22, 1.36)* | *0.98 (-1.39, 3.35)* | *-0.13 (-3.09, 2.82)* | **CCM** |

Note: * indicates P < 0.05, Network results are denoted in italics.

Abbreviations: CCM, Conventional compression moulding; CCI, Conventional injection moulding; CCA, Conventional autopolymerisation; MIL, CAD-CAM milled; TDP, Three-dimensional printed

**Supplementary Table 16.** League table showing the results of sensitivity analysis by excluding low quality studies: Flexural modulus.

| **CCI** | NA | NA | NA |
| --- | --- | --- | --- |
| *3.54 (0.97,6.12)** | **MIL** | NA | NA |
| *4.38 (1.47,7.28)** | *0.83 (-1.13,2.80)* | **TDP** | NA |
| *2.69 (0.14,5.24)** | *-0.85 (-2.59,0.88)* | *-1.69 (-3.39,0.02)* | **CCM** |

Note: * indicates P < 0.05, Network results are denoted in italics.

Abbreviations: CCI, Conventional injection moulding; MIL, CAD-CAM milled; TDP, Three-dimensional printed; CCM, Conventional compression moulding

**Supplementary Table 17**. League table showing the network and pairwise results for fracture toughness.

| **CCA** | NA | -0.04 (-0.27, 0.20) | NA | -0.14 (-0.22, -0.06) * |
| --- | --- | --- | --- | --- |
| *-0.57 (-4.71, 3.57)* | **CCI** | -0.24 (-0.49, 0.01) | NA | 0.46 (0.31, 0.61) * |
| *-0.30 (-3.30, 2.70)* | *0.27 (-2.81, 3.35)* | **MIL** | 0.14 (-0.77, 1.04) | 0.05 (-0.19, 0.29) |
| *-0.68 (-4.20, 2.84)* | *-0.11 (-3.70, 3.48)* | *-0.38 (-2.57, 1.82)* | **TDP** | -0.28 (-1.22, 0.65) |
| *0.09 (-2.92, 3.11)* | *0.66 (-2.44, 3.77)* | *0.39 (-1.31, 2.10)* | *0.77 (-1.42, 2.96)* | **CCM** |

Note: * indicates P < 0.05, Network results are denoted in italics.

Abbreviations: CCM, Conventional compression moulding; CCI, Conventional injection moulding; CCA, Conventional autopolymerisation; MIL, CAD-CAM milled; TDP, Three-dimensional printed

**Supplementary Table 18.** Results of network meta-analysis: Fracture toughness with SUCRA ranking.

| **Interventions** | **Fracture toughness** | | |
| --- | --- | --- | --- |
|  | **SMD (95% CI)** | **P** | **SUCRA rank (score)** |
| **TDP** | 0.77 (-1.42, 2.96) | 0.49 | 1 (64.1) |
| **CCI** | 0.66 (-2.44, 3.77) | 0.675 | 2 (57.8) |
| **MIL** | 0.39 (-1.31, 2.10) | 0.650 | 3 (50.3) |
| **CCA** | 0.09 (-2.92, 3.11) | 0.951 | 4 (43) |
| **CCM** | Reference | | 5 (34.9) |

Abbreviations: CCM, conventional compression moulding; CCI, conventional injection moulding; CCA, conventional autopolymerisation; MIL, CAD-CAM milled; TDP, three-dimensional printed

**Supplementary Table 19**. League table showing the network and pairwise results for yield point.

| CCI | -0.85 (-1.43, -0.27) * | NA | -3.69 (-4.62, -2.77) * |
| --- | --- | --- | --- |
| *-2.16 (-8.38, 4.05)* | MIL | 0.96 (0.16, 1.76) * | -1.71 (-8.78, 5.37) |
| *-1.24 (-10.34, 7.86)* | *0.93 (-5.72, 7.58)* | TDP | NA |
| *-2.91 (-9.14, 3.33)* | *-0.74 (-5.49, 4.01)* | *-1.67 (-9.84, 6.50)* | CCM |

Note: * indicates P < 0.05, Network results are denoted in italics.

Abbreviations: CCM, Conventional compression moulding; CCI, Conventional injection moulding; MIL, CAD-CAM milled; TDP, Three-dimensional printed

**Supplementary Table 20.** Results of network meta-analysis: Yield point with SUCRA ranking.

| **Interventions** | **Yield point** | | |
| --- | --- | --- | --- |
|  | **SMD (95% CI)** | **P** | **SUCRA rank (score)** |
| **CCM** | Reference | | 1 (69.6) |
| **MIL** | -0.74 (-5.49, 4.01) | 0.760 | 2 (58.5) |
| **TDP** | -1.67 (-9.84, 6.50) | 0.689 | 3 (45.6) |
| **CCI** | -2.91 (-9.14, 3.33) | 0.361 | 4 (26.3) |

Abbreviations: CCM, conventional compression moulding; CCI, conventional injection moulding; MIL, CAD-CAM milled; TDP, three-dimensional printed

**Supplementary Table 21**. League table showing the network and pairwise results for strain at yield point.

| **MIL** | -0.35 (-1.11, 0.42) | -4.46 (-6.98, -1.95) * |
| --- | --- | --- |
| *-0.34 (-1.10, 0.43) ** | **TDP** | NA |
| *-4.03 (-6.70, -1.36) ** | *-3.69 (-6.47, -0.91) ** | **CCM** |

Note: * indicates P < 0.05, Network results are denoted in italics.

Abbreviations: CCM, Conventional compression moulding; MIL, CAD-CAM milled; TDP, Three-dimensional printed

**Supplementary Table 22.** Results of network meta-analysis: Strain at yield point with SUCRA ranking.

| **Interventions** | **Strain at yield point** | | |
| --- | --- | --- | --- |
|  | **SMD (95% CI)** | **P** | **SUCRA rank (score)** |
| **CCM** | Reference | | 1 (99.8) |
| **TDP** | -3.69 (-6.47, -0.91) | 0.009* | 2 (41.1) |
| **MIL** | -4.03 (-6.70, -1.36) | 0.003* | 3 (9.1) |

Note: presence of (*) and texts in red indicate intervention statistically significant (P<.05).

Abbreviations: CCM, conventional compression moulding; MIL, CAD-CAM milled; TDP, three-dimensional printed

**Supplementary Table 23**. League table showing the network and pairwise results for toughness.

| **MIL** | 0.97 (0.17, 1.77) * | 7.61 (3.68, 11.54) * |
| --- | --- | --- |
| *0.94 (0.14, 1.74) ** | **TDP** | NA |
| *6.87 (2.67, 11.06) ** | *5.93 (1.65, 10.21) ** | **CCM** |

Note: * indicates P < 0.05, Network results are denoted in italics.

Abbreviations: CCM, Conventional compression moulding; MIL, CAD-CAM milled; TDP, Three-dimensional printed

**Supplementary Table 24.** Results of network meta-analysis: Toughness with SUCRA ranking.

| **Interventions** | **Toughness** | | |
| --- | --- | --- | --- |
|  | **SMD (95% CI)** | **P** | **SUCRA rank (score)** |
| **MIL** | 6.87 (2.67, 11.06) | 0.001* | 1 (99.4) |
| **TDP** | 5.93 (1.65, 10.21) | 0.007* | 2 (50.3) |
| **CCM** | Reference | | 3 (0.2) |

Note: presence of (*) and texts in red indicate intervention statistically significant (P<.05).

Abbreviations: CCM, conventional compression moulding; MIL, CAD-CAM milled; TDP, three-dimensional printed

**Supplementary Table 25**. Results of node-splitting: Flexural strength.

| Side | Direct |  | Indirect |  | Difference |  |  |
| --- | --- | --- | --- | --- | --- | --- | --- |
|  | Coef. | Std. Err. | Coef. | Std. Err. | Coef. | Std. Err. | **P>z** |
| C D * | 1.332468 | 0.5322094 | -3.41966 | 1.392893 | 4.752128 | 1.491373 | **0.058** |
| C E | -1.333457 | 0.5927073 | 1.308289 | 1.332016 | -2.641746 | 1.455431 | **0.07** |
| A C * | -1.74347 | 2.658298 | 5.471284 | 4.80652 | -7.214754 | 5.53388 | **0.192** |
| A D * | 2.421487 | 2.668792 | -4.793267 | 4.789042 | 7.214754 | 5.53388 | **0.192** |
| B C | 0.8630423 | 0.9051379 | 2.774722 | 1.592158 | -1.911679 | 1.824311 | **0.295** |
| B D * | 2.207184 | 0.8806991 | 1.247417 | 1.868538 | 0.9597671 | 2.072272 | **0.643** |
| B E | 0.9359897 | 1.223061 | -0.060158 | 1.152262 | 0.9961475 | 1.679251 | **0.553** |
| D E | -0.836558 | 0.5974026 | -4.582315 | 1.195903 | 3.745757 | 1.336228 | **0.055** |

Note: * indicates that findings are in logarithmic form, A p-value < 0.05 suggests significant inconsistency between the direct and indirect estimates for a specific treatment comparison, indicating that direct and indirect evidence may not agree for that comparison.

Abbreviations: A=Conventional autopolymerisation; B=Conventional injection moulding; C=Conventional compresion moulding; D=CAD-CAM milled; E=Three-dimensional printed

**Supplementary Table 26**. Results of node-splitting: Hardness.

| Side | Direct |  | Indirect |  | Difference |  |  |
| --- | --- | --- | --- | --- | --- | --- | --- |
|  | Coef. | Std. Err. | Coef. | Std. Err. | Coef. | Std. Err. | **P>z** |
| B C | 0.613088 | 1.592887 | 2.495098 | 3.132751 | -1.88201 | 3.513728 | **0.592** |
| B D | -2.72895 | 1.554622 | 1.149354 | 2.927565 | -3.8783 | 3.315277 | **0.242** |
| A B | 1.734789 | 2.193631 | 6.371776 | 3.455458 | -4.63699 | 4.095358 | **0.258** |
| A C * | 3.59397 | 2.019777 | 6.526229 | 4.556652 | -2.93226 | 4.981604 | **0.556** |
| A D | 2.61582 | 2.573277 | -0.5978 | 2.88786 | 3.213621 | 3.862586 | **0.405** |
| C D | -3.03955 | 1.608726 | -2.29169 | 3.121082 | -0.74785 | 3.512619 | **0.831** |

Note: * indicates that findings are in logarithmic form, A p-value < 0.05 suggests significant inconsistency between the direct and indirect estimates for a specific treatment comparison, indicating that direct and indirect evidence may not agree for that comparison.

Abbreviations: A=Conventional injection moulding; B=Conventional compresion moulding; C=CAD-CAM milled; D=Three-dimensional printed

**Supplementary Table 27**. Results of node-splitting: Impact strength.

| Side | Direct |  | Indirect |  | Difference |  |  |
| --- | --- | --- | --- | --- | --- | --- | --- |
|  | Coef. | Std. Err. | Coef. | Std. Err. | Coef. | Std. Err. | **P>z** |
| C D | 1.994486 | 3.323318 | 2.428551 | 5.138121 | -0.43406 | 6.119162 | **0.943** |
| C E * | -0.83715 | 2.035805 | -4.54682 | 4.940206 | 3.709666 | 5.342803 | **0.487** |
| A C * | -11.9545 | 3.945169 | 2.375857 | 7.50046 | -14.3303 | 8.446692 | **0.09** |
| A E * | -7.37191 | 3.864458 | -21.7022 | 7.625475 | 14.33031 | 8.446692 | **0.09** |
| B C | -1.8046 | 4.311014 | -7.87811 | 4.254944 | 6.073513 | 6.058389 | **0.316** |
| B D * | -2.45749 | 3.297174 | -6.18018 | 11.09799 | 3.722684 | 11.5697 | **0.748** |
| B E | -10.0209 | 4.133628 | -2.27275 | 4.222989 | -7.74811 | 5.90842 | **0.19** |
| D E | -3.76444 | 4.696915 | -3.33001 | 3.92602 | -0.43444 | 6.119366 | **0.943** |

Note: * indicates that findings are in logarithmic form, A p-value < 0.05 suggests significant inconsistency between the direct and indirect estimates for a specific treatment comparison, indicating that direct and indirect evidence may not agree for that comparison.

Abbreviations: A=Conventional autopolymerisation; B=Conventional injection moulding; C=Conventional compresion moulding; D=CAD-CAM milled; E=Three-dimensional printed

**Supplementary Table 28**. Results of node-splitting: Elastic modulus.

| Side | Direct |  | Indirect |  | Difference |  |  |
| --- | --- | --- | --- | --- | --- | --- | --- |
|  | Coef. | Std. Err. | Coef. | Std. Err. | Coef. | Std. Err. | **P>z** |
| C D * | 0.908162 | 2.390989 | -4.84518 | 7.57149 | 5.753344 | 7.94003 | **0.469** |
| C E | -5.59433 | 2.891008 | -3.49619 | 6.636566 | -2.09814 | 7.218057 | **0.771** |
| A C * | 0.157432 | 5.634052 | 1.630411 | 11.09683 | -1.47298 | 12.44498 | **0.906** |
| A D * | 1.150571 | 5.62742 | -0.32241 | 11.10692 | 1.472978 | 12.44499 | **0.906** |
| B C * | -1.97998 | 5.462714 | -8.65359 | 10.78625 | 6.673613 | 12.08428 | **0.581** |
| B D * | -4.31994 | 5.470619 | 2.353664 | 10.77422 | -6.67361 | 12.08427 | **0.581** |
| D E | -5.86909 | 2.917132 | -4.55194 | 6.672819 | -1.31715 | 7.264911 | **0.856** |

Note: * indicates that findings are in logarithmic form, A p-value < 0.05 suggests significant inconsistency between the direct and indirect estimates for a specific treatment comparison, indicating that direct and indirect evidence may not agree for that comparison.

Abbreviations: A=Conventional autopolymerisation; B=Conventional injection moulding; C=Conventional compresion moulding; D=CAD-CAM milled; E=Three-dimensional printed

**Supplementary Table 29**. Results of node-splitting: Flexural modulus.

| Side | Direct |  | Indirect |  | Difference |  |  |
| --- | --- | --- | --- | --- | --- | --- | --- |
|  | Coef. | Std. Err. | Coef. | Std. Err. | Coef. | Std. Err. | **P>z** |
| C D * | 1.396688 | 1.358749 | -1.18519 | 3.127437 | 2.581881 | 3.41683 | **0.45** |
| C E | -1.0733 | 2.045555 | 1.129355 | 2.366956 | -2.20265 | 3.127818 | **0.481** |
| A C * | -2.38071 | 3.630355 | -4.63588 | 6.829023 | 2.255175 | 7.719497 | **0.77** |
| A D * | -2.38071 | 3.630354 | -0.12553 | 6.829021 | -2.25518 | 7.719495 | **0.77** |
| B C | 1.658677 | 2.563989 | 3.652147 | 3.110409 | -1.99347 | 3.986673 | **0.617** |
| B D * | 3.13785 | 2.177212 | 4.809282 | 4.483741 | -1.67143 | 4.977451 | **0.737** |
| B E | 4.902794 | 3.488185 | 0.823993 | 2.632918 | 4.078801 | 4.370101 | **0.351** |
| D E | -0.76017 | 1.81334 | -1.97986 | 2.806499 | 1.219699 | 3.340455 | **0.715** |

Note: * indicates that findings are in logarithmic form, A p-value < 0.05 suggests significant inconsistency between the direct and indirect estimates for a specific treatment comparison, indicating that direct and indirect evidence may not agree for that comparison.

Abbreviations: A=Conventional autopolymerisation; B=Conventional injection moulding; C=Conventional compresion moulding; D=CAD-CAM milled; E=Three-dimensional printed

**SUPPLEMENTARY FIGURES**

**Supplementary Figure 1.** Forest plot for flexural strength.


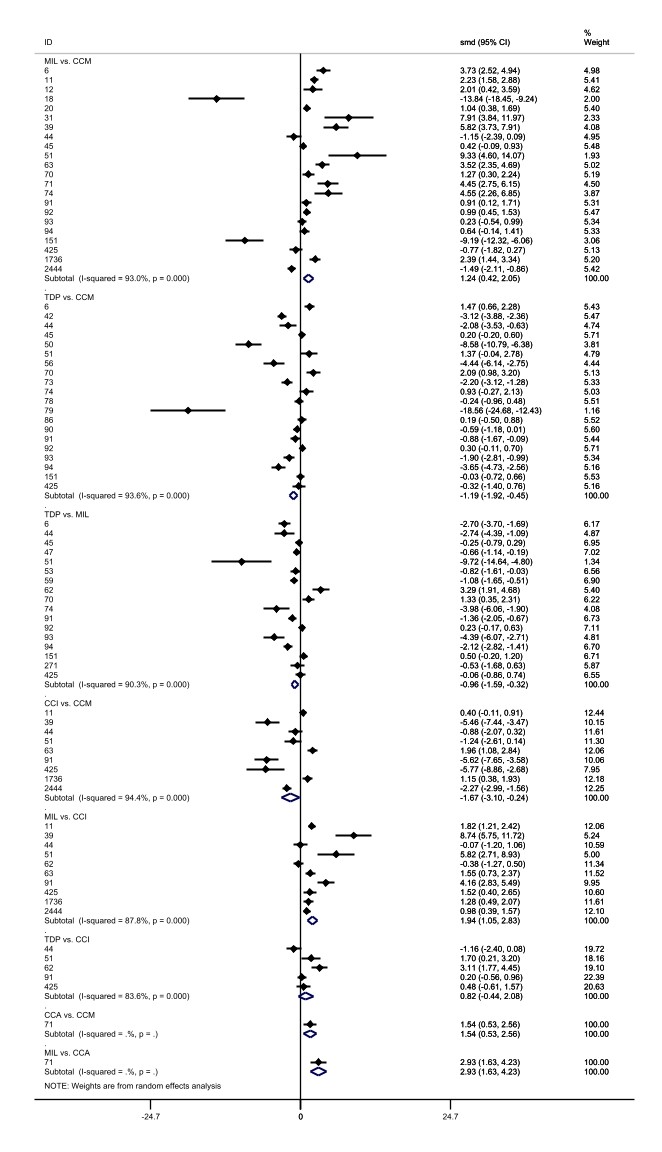

Abbreviations: CCM, Conventional compression moulding; CCI, Conventional injection moulding; CCA, Conventional autopolymerisation; MIL, CAD-CAM milled; TDP, Three-dimensional printed

**Supplementary Figure 2.** Comparison-adjusted funnel plot for flexural strength.


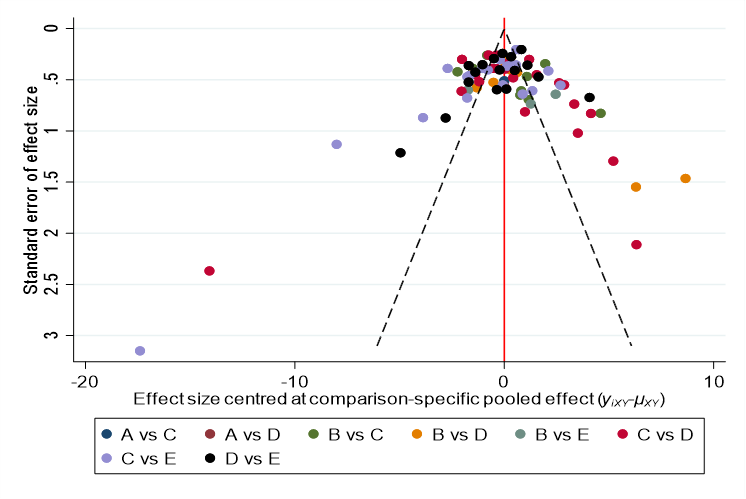


Abbreviations: A=Conventional autopolymerisation; B=Conventional injection moulding; C=Conventional compression moulding; D=CAD-CAM milled; E=Three-dimensional printed

**Supplementary Figure 3.** Forest plot for hardness.


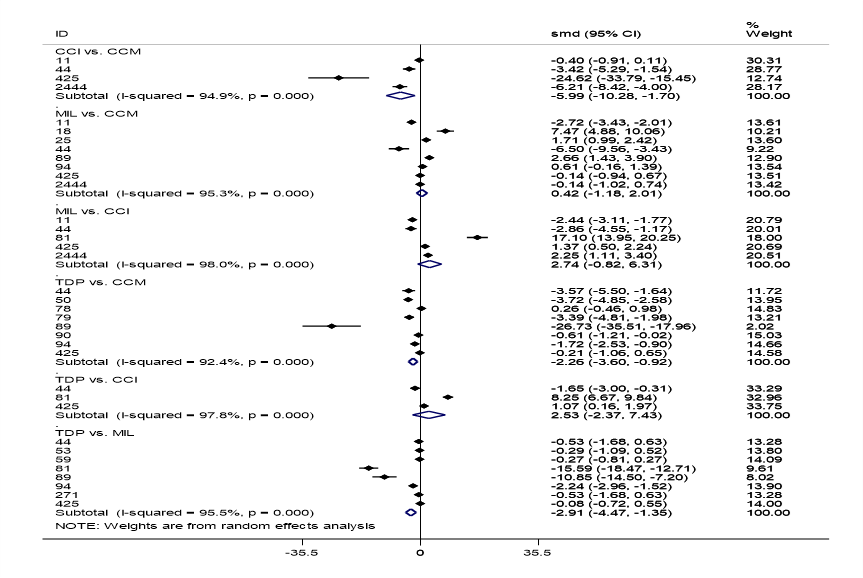


Abbreviations: CCM, Conventional compression moulding; CCI, Conventional injection moulding; MIL, CAD-CAM milled; TDP, Three-dimensional printed

**Supplementary Figure 4.** Comparison-adjusted funnel plot for hardness.


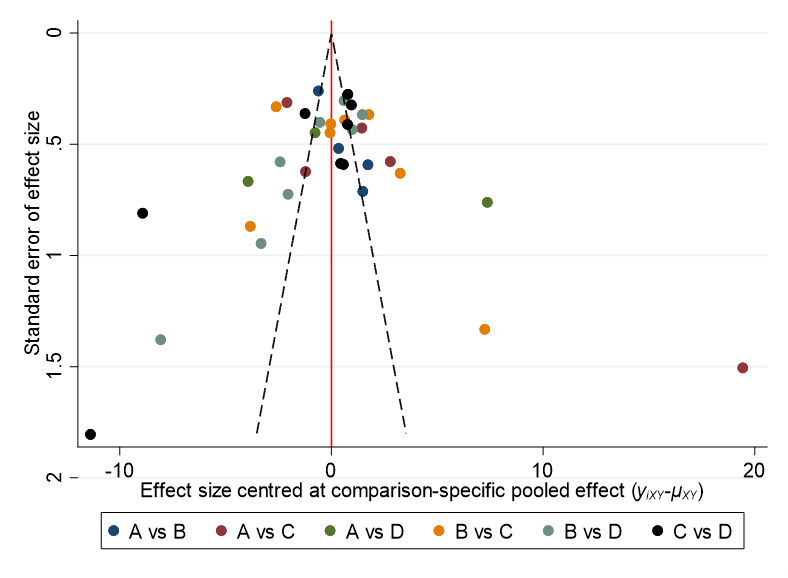


Abbreviations: A= Conventional injection moulding; B=Conventional compression moulding; C=CAD-CAM milled; D=Three-dimensional printed

**Supplementary Figure 5.** Forest plot for impact strength.


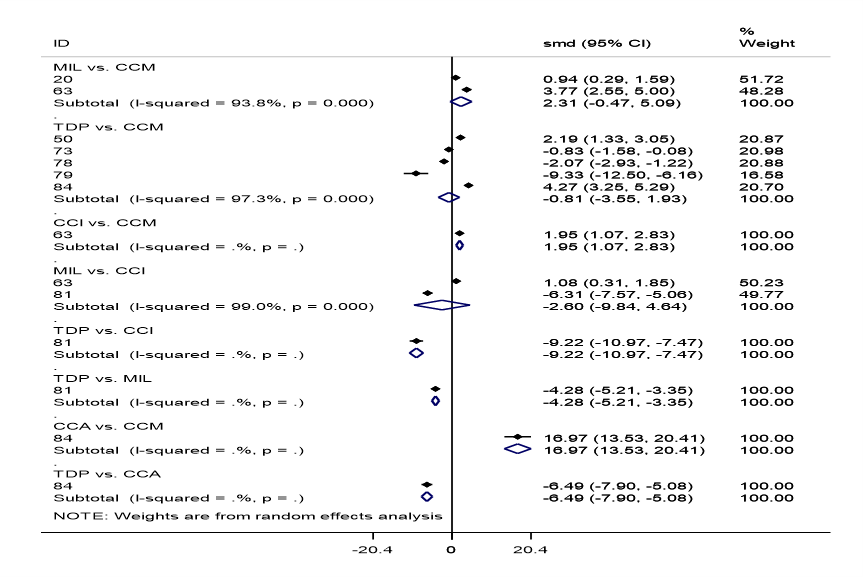


Abbreviations: CCM, Conventional compression moulding; CCI, Conventional injection moulding; CCA, Conventional auto polymerisation; MIL, CAD-CAM milled; TDP, Three-dimensional printed

**Supplementary Figure 6.** Comparison-adjusted funnel plot for impact strength.


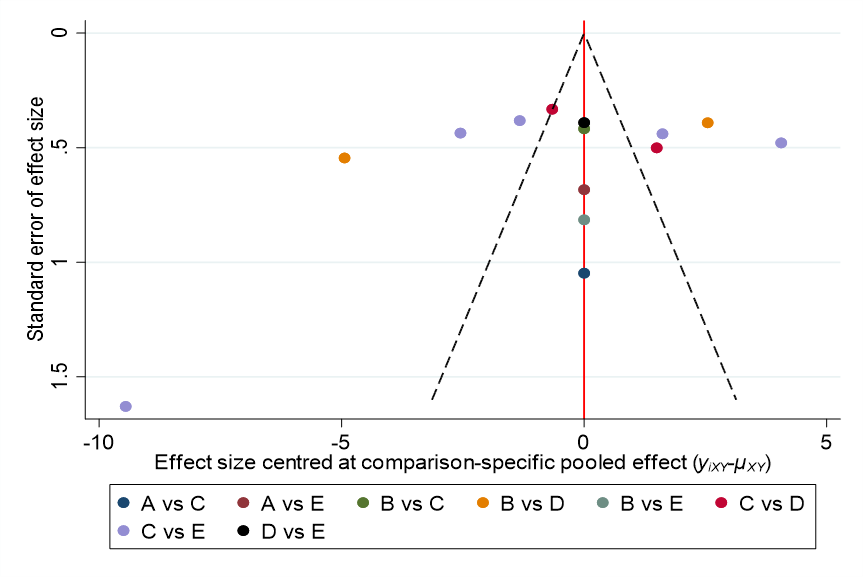


Abbreviations: A= Conventional autopolymerisation; B= Conventional injection moulding; C= Conventional compression moulding; D=CAD-CAM milled; E=Three-dimensional printed

**Supplementary Figure 7.** Forest plot for elastic modulus.


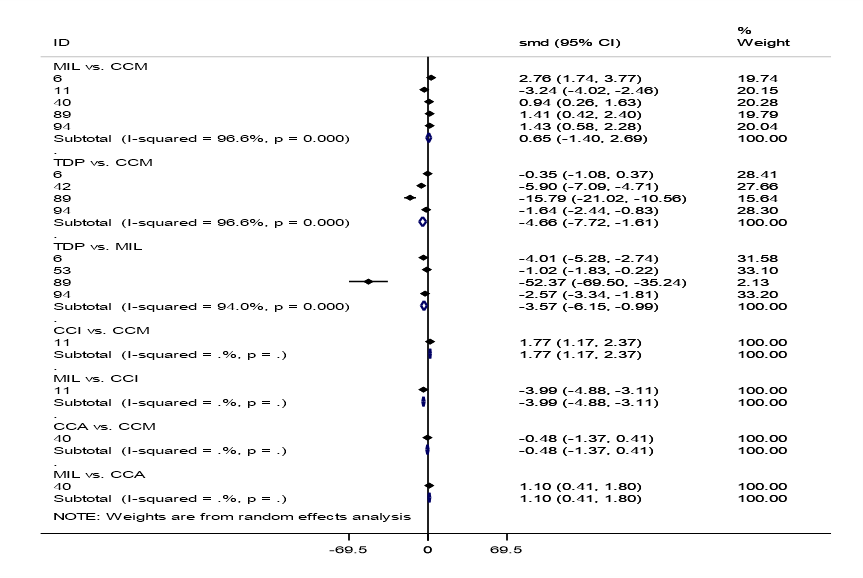


Abbreviations: CCM, Conventional compression moulding; CCI, Conventional injection moulding; CCA, Conventional auto polymerisation; MIL, CAD-CAM milled; TDP, Three-dimensional printed

**Supplementary Figure 8.** Comparison-adjusted funnel plot for elastic modulus.


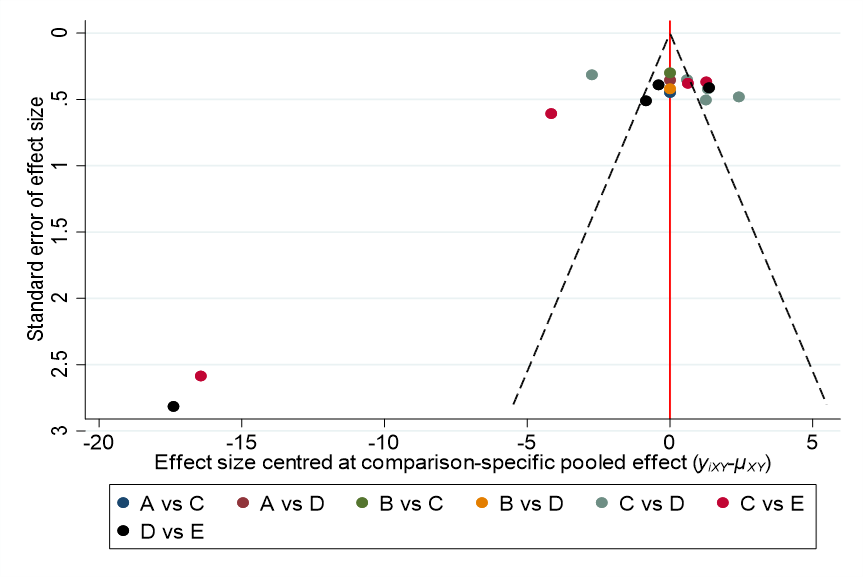


Abbreviations: A=Conventional autopolymerisation; B=Conventional injection moulding; C=Conventional compresion moulding; D=CAD-CAM milled; E=Three-dimensional printed

**Supplementary Figure 9.** Forest plot for flexural modulus.


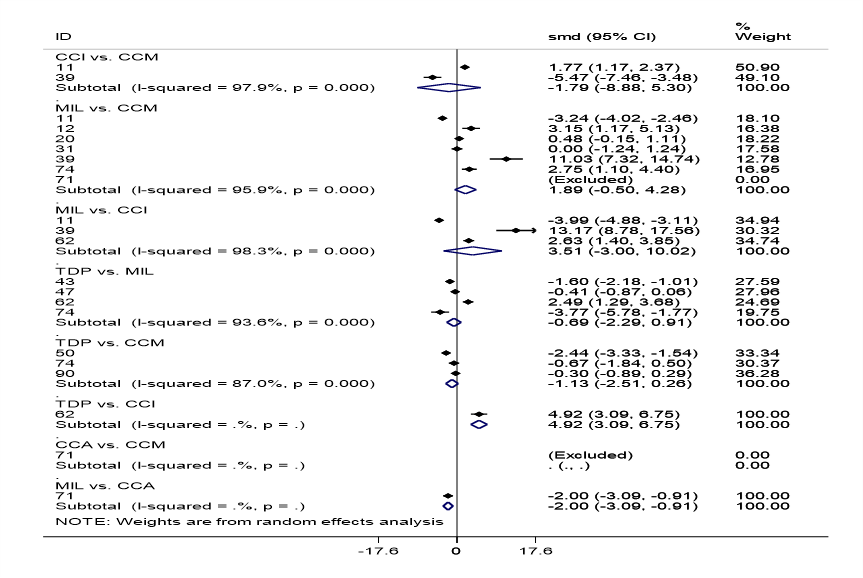


Abbreviations: CCM, Conventional compression moulding; CCI, Conventional injection moulding; CCA, Conventional auto polymerisation; MIL, CAD-CAM milled; TDP, Three-dimensional printed

**Supplementary Figure 10.** Comparison-adjusted funnel plot for flexural modulus.


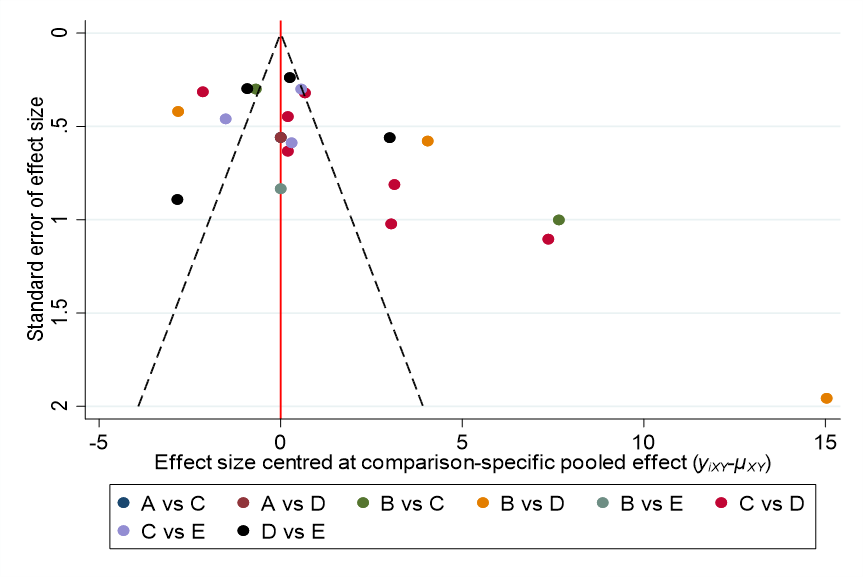


Abbreviations: A=Conventional autopolymerisation; B=Conventional injection moulding; C=Conventional compression moulding; D=CAD-CAM milled; E=Three-dimensional printed

**Supplementary Figure 11A.** Network plot for fracture toughness.


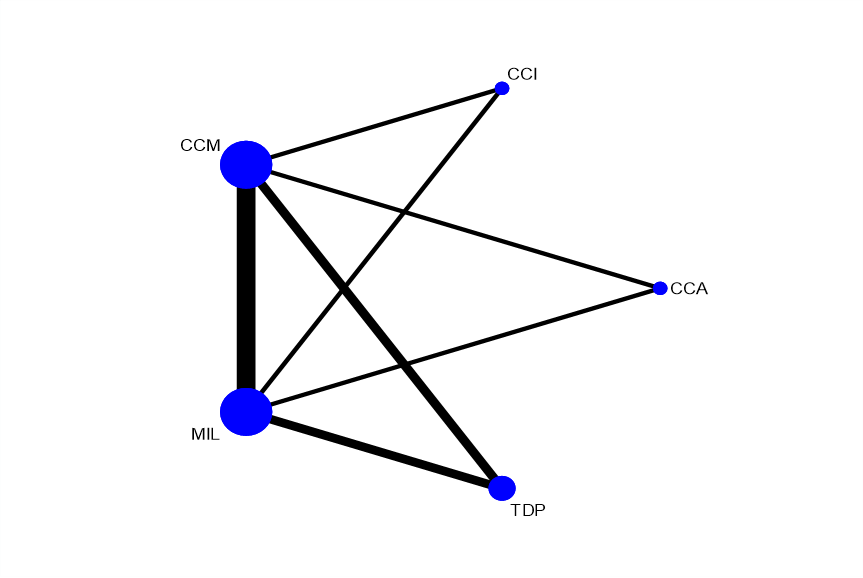


Abbreviations: CCM, Conventional compression moulding; CCI, Conventional injection moulding; CCA, Conventional auto polymerisation; MIL, CAD-CAM milled; TDP, Three-dimensional printed

**Supplementary Figure 11B.** SUCRA ranking curve in fracture toughness.


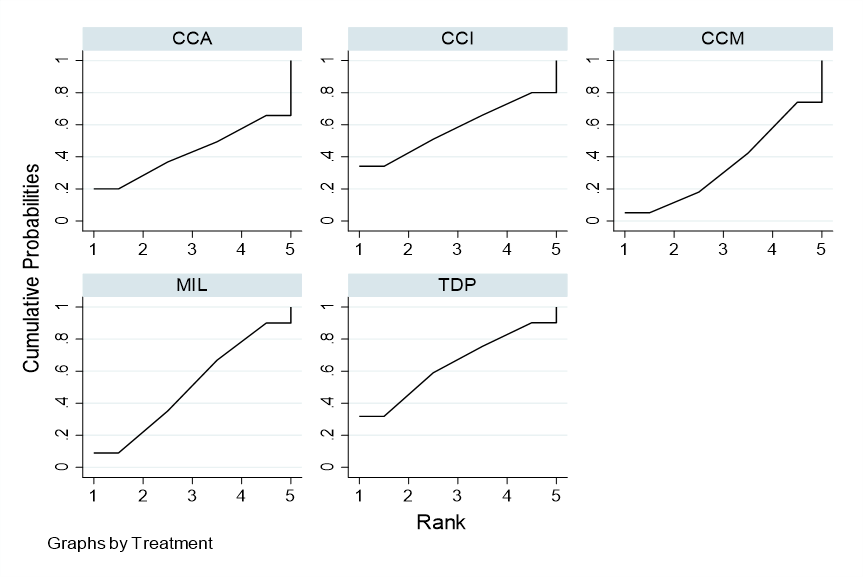


Abbreviations: CCM, Conventional compression moulding; CCI, Conventional injection moulding; CCA, Conventional auto polymerisation; MIL, CAD-CAM milled; TDP, Three-dimensional printed

**Supplementary Figure 12.** Forest plot for fracture toughness.


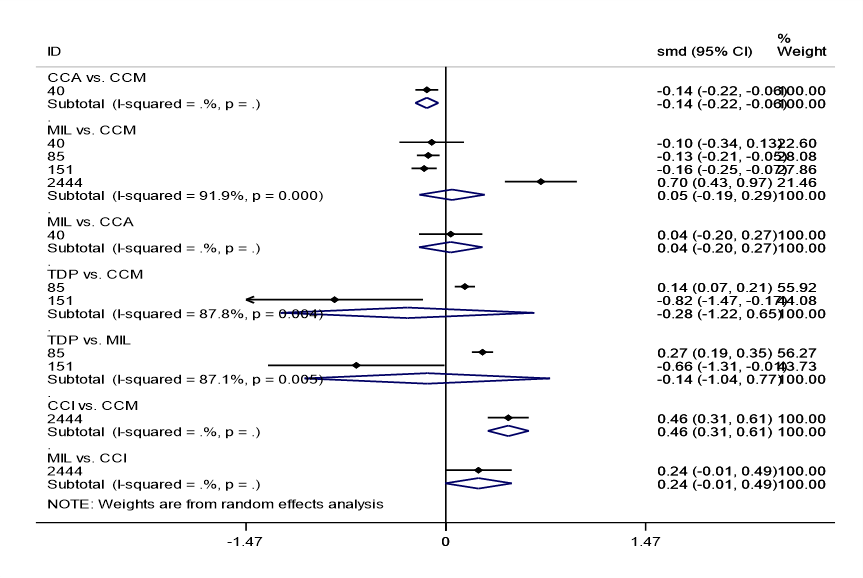


Abbreviations: CCM, Conventional compression moulding; CCI, Conventional injection moulding; CCA, Conventional auto polymerisation; MIL, CAD-CAM milled; TDP, Three-dimensional printed

**Supplementary Figure 13.** Comparison-adjusted funnel plot for fracture toughness.


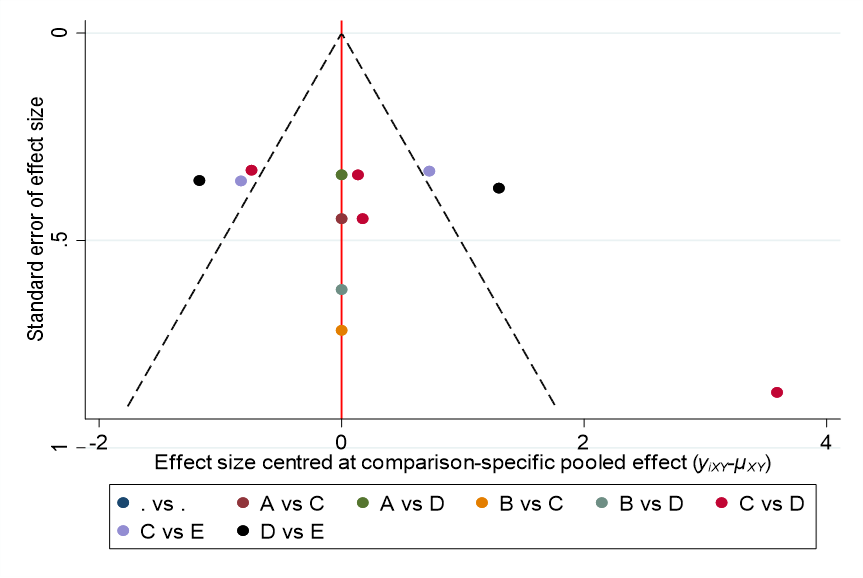


Abbreviations: A=Conventional autopolymerisation; B=Conventional injection moulding; C=Conventional compression moulding; D=CAD-CAM milled; E=Three-dimensional printed

**Supplementary Figure 14A.** Network plot for yield point.


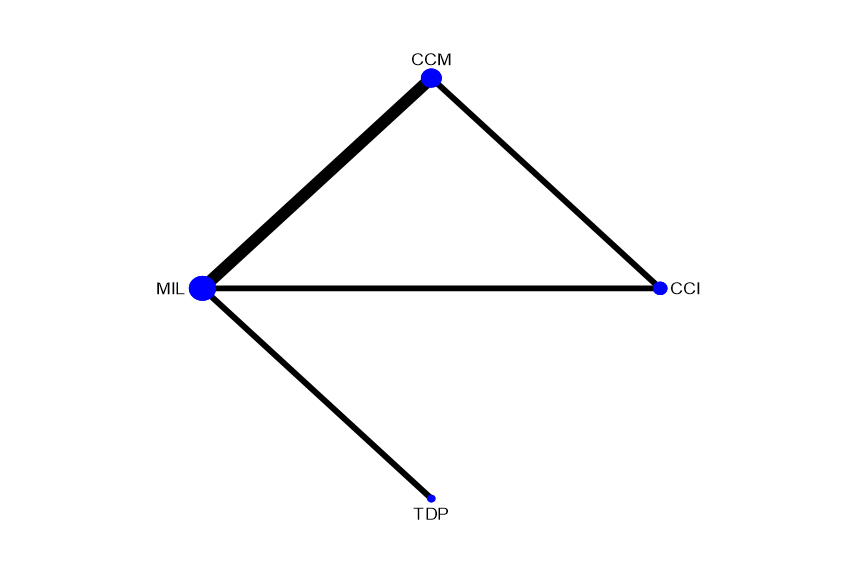
Abbreviations: CCM, Conventional compression moulding; CCI, Conventional injection moulding; MIL, CAD-CAM milled; TDP, Three-dimensional printed

**Supplementary Figure 14B.** SUCRA ranking curve in yield point.


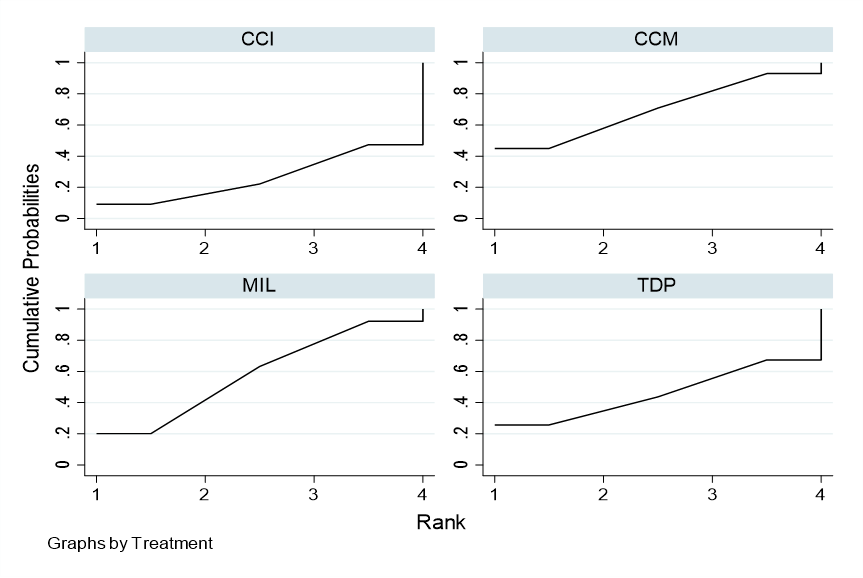


Abbreviations: CCM, Conventional compression moulding; CCI, Conventional injection moulding; MIL, CAD-CAM milled; TDP, Three-dimensional printed

**Supplementary Figure 15.** Forest plot for yield point.


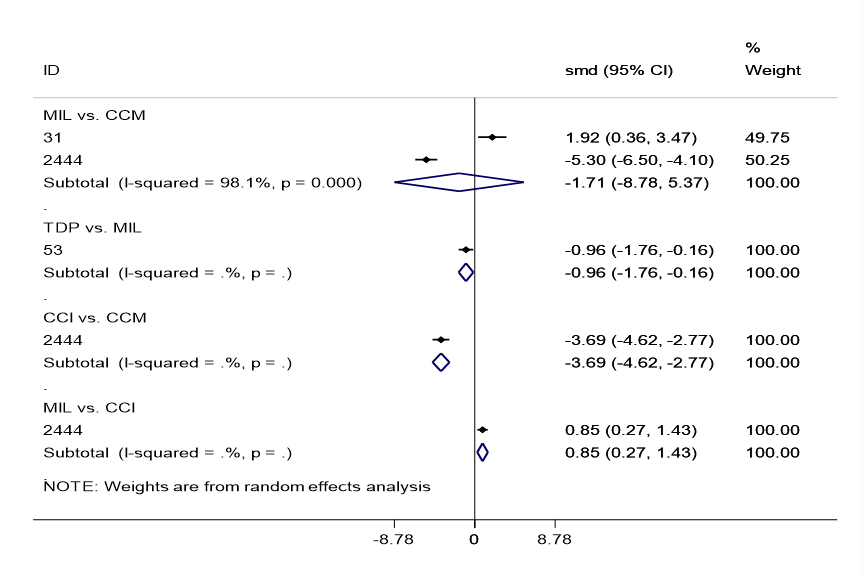


Abbreviations: CCM, Conventional compression moulding; CCI, Conventional injection moulding; MIL, CAD-CAM milled; TDP, Three-dimensional printed

**Supplementary Figure 16.** Comparison-adjusted funnel plot for yield point.


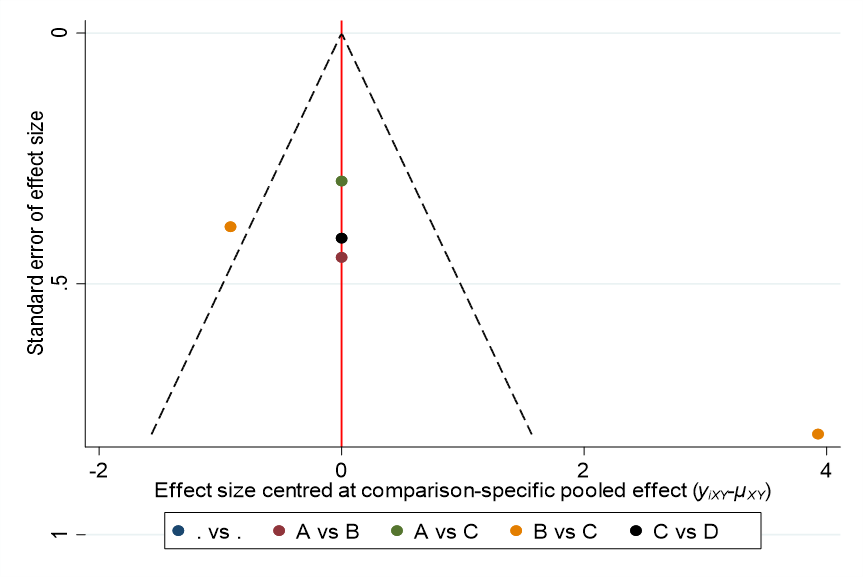


Abbreviations: A=Conventional injection moulding; B=Conventional compression moulding; C=CAD-CAM milled; D=Three-dimensional printed

**Supplementary Figure 17A.** Network plot for strain at yield point.


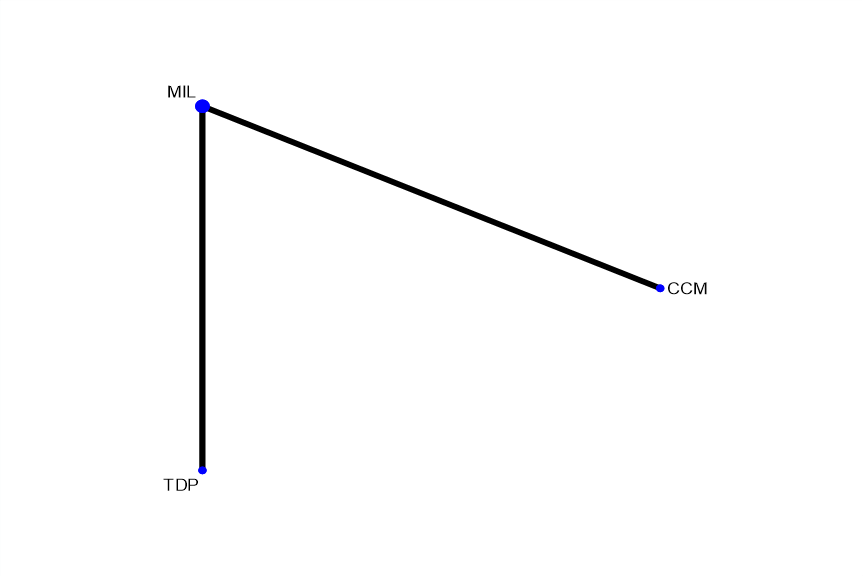


Abbreviations: CCM, Conventional compression moulding; MIL, CAD-CAM milled; TDP, Three-dimensional printed

**Supplementary Figure 17B.** SUCRA ranking curve in strain at yield point.


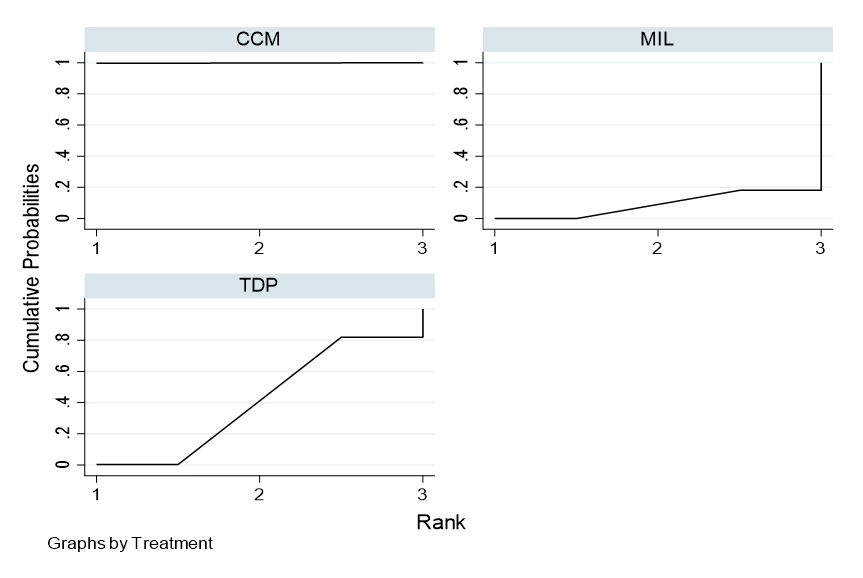


Abbreviations: CCM, Conventional compression moulding; MIL, CAD-CAM milled; TDP, Three-dimensional printed

**Supplementary Figure 18.** Forest plot for strain at yield point.


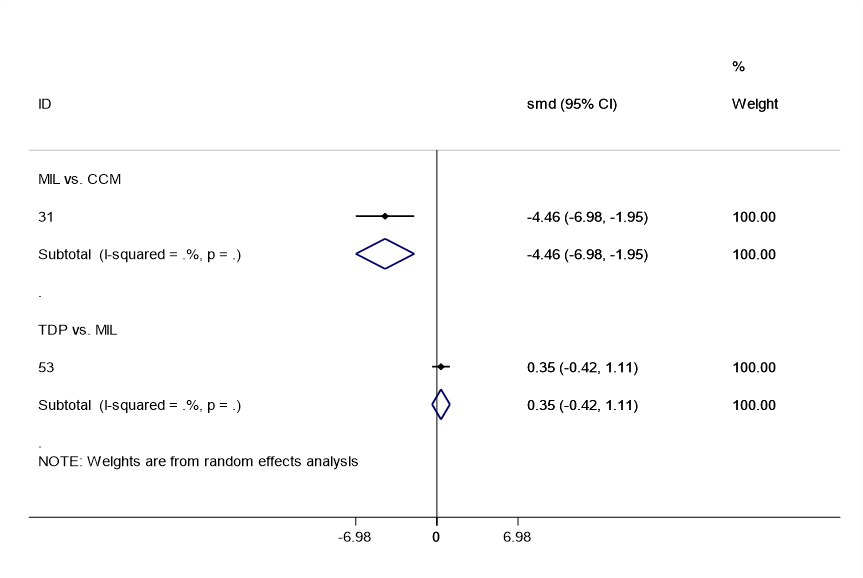


Abbreviations: CCM, Conventional compression moulding; MIL, CAD-CAM milled; TDP, Three-dimensional printed

**Supplementary Figure 19.** Comparison-adjusted funnel plot for strain at yield point.


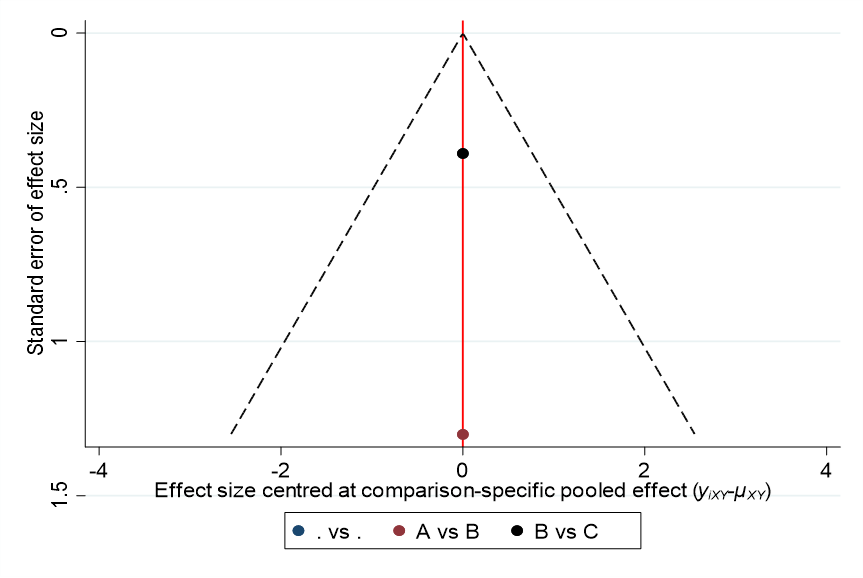


Abbreviations: A=Conventional compression moulding; B=CAD-CAM milled; C=Three-dimensional printed

**Supplementary Figure 20A.** Network plot for toughness.


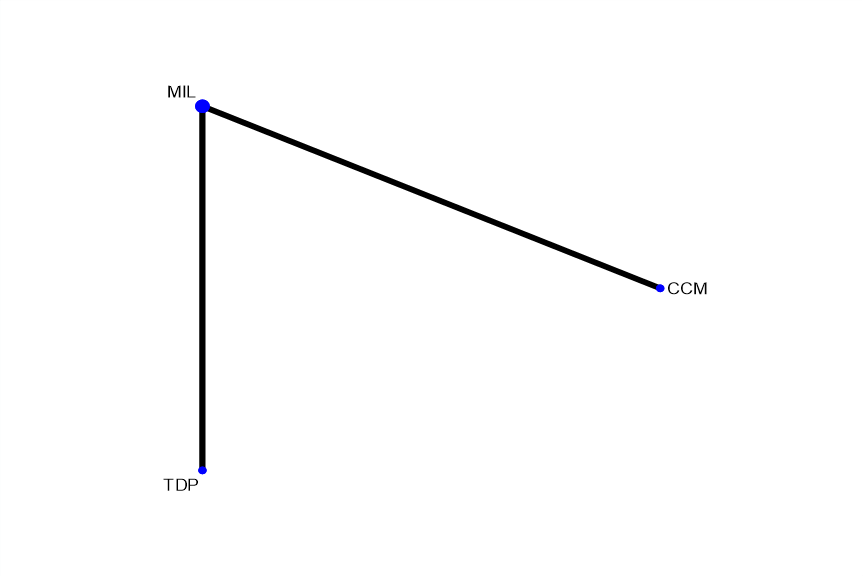
Abbreviations: CCM, Conventional compression moulding; MIL, CAD-CAM milled; TDP, Three-dimensional printed

**Supplementary Figure 20B.** SUCRA ranking curve in toughness.


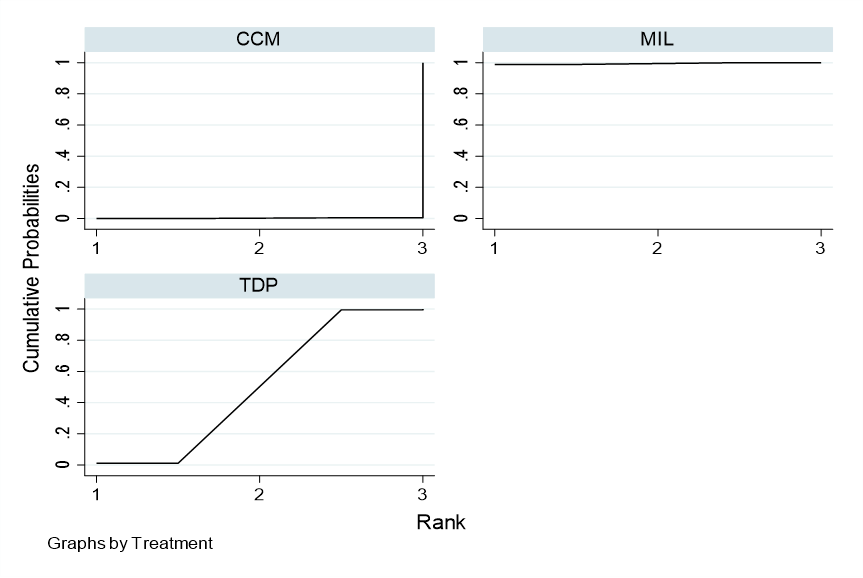


Abbreviations: CCM, Conventional compression moulding; MIL, CAD-CAM milled; TDP, Three-dimensional printed

**Supplementary Figure 21.** Forest plot for toughness.


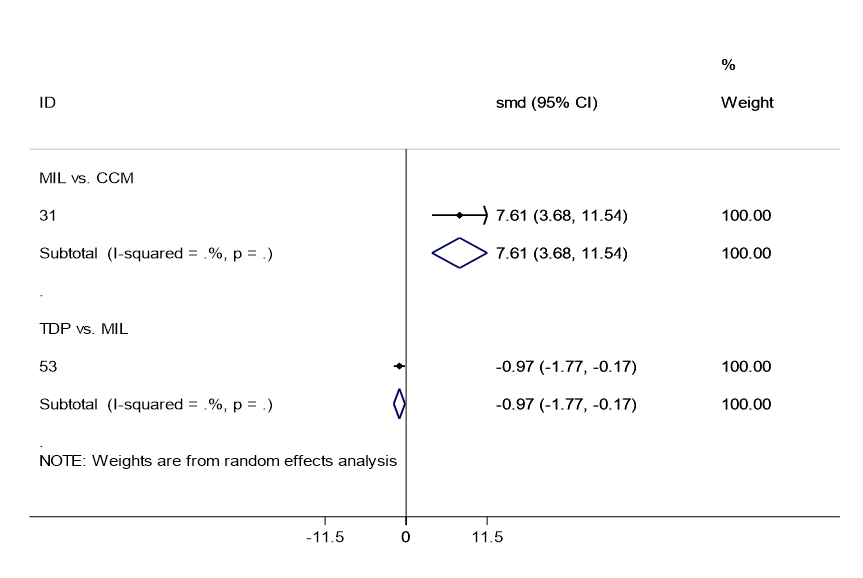


Abbreviations: CCM, Conventional compression moulding; MIL, CAD-CAM milled; TDP, Three-dimensional printed

**Supplementary Figure 22.** Comparison-adjusted funnel plot for toughness.


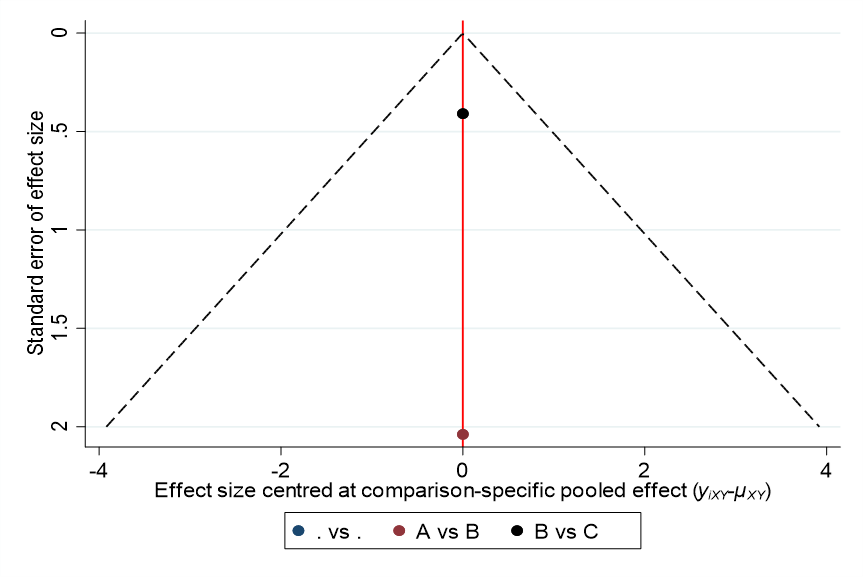


Abbreviations: A=Conventional compression moulding; B=CAD-CAM milled; C=Three-dimensional printed
